# Combination therapy with saxagliptin and vitamin D for the preservation of β-cell function in adult-onset type 1 diabetes: a multi-center, randomized, controlled trial

**DOI:** 10.1038/s41392-023-01369-9

**Published:** 2023-04-20

**Authors:** Xiang Yan, Xia Li, Bingwen Liu, Jiaqi Huang, Yufei Xiang, Yuhang Hu, Xiaohan Tang, Ziwei Zhang, Gan Huang, Zhiguo Xie, Houde Zhou, Zhenqi Liu, Xiangbing Wang, Richard David Leslie, Zhiguang Zhou

**Affiliations:** 1 National Clinical Research Center for Metabolic Diseases, Key Laboratory of Diabetes Immunology (Central South University), Ministry of Education, and Department of Metabolism and Endocrinology, The Second Xiangya Hospital of Central South University, Changsha, Hunan China; 2 Division of Endocrinology and Metabolism, Department of Medicine, University of Virginia Health System, Charlottesville, VA USA; 3 Division of Endocrinology, Metabolism and Nutrition, Rutgers University-Robert Wood Johnson Medical School, New Brunswick, NJ USA; 4 Centre for Immunobiology, Blizard Institute, Queen Mary University of London, London, UK

**Keywords:** Endocrine system and metabolic diseases, Clinical trial design

## Abstract

Disease modifying therapies aiming to preserve β-cell function in patients with adult-onset autoimmune type 1 diabetes are lacking. Here, we conducted a multi-centre, randomized, controlled trial to assess the β-cell preservation effects of saxagliptin alone and saxagliptin combined with vitamin D as adjunctive therapies in adult-onset autoimmune type 1 diabetes. In this 3-arm trial, 301 participants were randomly assigned to a 24-month course of the conventional therapy (metformin with or without insulin) or adjunctive saxagliptin or adjunctive saxagliptin plus vitamin D to the conventional therapy. The primary endpoint was the change from baseline to 24 months in the fasting C-peptide. The secondary endpoints included the area under the concentration-time curve (AUC) for C-peptide level in a 2-h mixed-meal tolerance test, glycemic control, total daily insulin use and safety, respectively. The primary endpoint was not achieved in saxagliptin plus vitamin D group (*P* = 0.18) and saxagliptin group (*P* = 0.26). However, compared with the conventional therapy, 2-h C-peptide AUC from 24 months to baseline decreased less with saxagliptin plus vitamin D (-276 pmol/L vs. -419 pmol/L; *P* = 0.01), and not to the same degree with saxagliptin alone (-314 pmol/L; *P* = 0.14). Notably, for participants with higher glutamic acid decarboxylase antibody (GADA) levels, the decline of β-cell function was much lower in saxagliptin plus vitamin D group than in the conventional therapy group (*P* = 0.001). Insulin dose was significantly reduced in both active treatment groups than in the conventional therapy group despite all groups having similar glycemic control. In conclusion, the combination of saxagliptin and vitamin D preserves pancreatic β-cell function in adult-onset autoimmune type 1 diabetes, an effect especially efficacious in individuals with higher GADA levels. Our results provide evidence for a novel adjunct to insulin and metformin as potential initial treatment for adult-onset type 1 diabetes. (ClinicalTrials.gov identifier: NCT02407899).

## Introduction

Autoimmune type 1 diabetes, mediated through immune perturbations, is charactered by progressive destruction of β-cells, and eventually a lifelong dependence on insulin therapy.^1^ Type 1 diabetes is recognised as a heterogenous disease and a feature of that heterogeneity is that adult-onset type 1 diabetes is immunogenetically distinct from the childhood-onset disease, with a less severe presentation and a more chronic course of metabolic dysfunction.^2^ Further, adult-onset type 1 diabetes is the commonest form of the disease and often presents without insulin-dependence leading to frequent misclassification as adult-onset type 2 diabetes; those cases initially non-insulin requiring with diabetes-associated autoantibodies have been called latent autoimmune diabetes of adults (LADA).^2^ These issues with misdiagnosis have led to a recent consensus statement about the nature and detection of adult-onset type 1 diabetes and the challenges it poses.^2,3^ Even more uncertain is the best approach to identification and management of such cases. Here we attempt to resolve that dilemma in part by reporting the first sufficiently powered study of adult-onset type 1 diabetes, including LADA, using dipeptidyl peptidase 4 (DPP-4) inhibitor and vitamin D as an adjunct to insulin and metformin.

So-called LADA, the most common subtype of adult-onset type 1 diabetes, affects around 10 million people in China, and it has been estimated that the incidence is gradually increasing worldwide.^4–6^ Most studies in epidemiology of adult-onset type 1 diabetes identified a slight association with male predominance.^7^ Appropriate treatment for such cases is vital given that autoimmune adult-onset diabetes, including LADA, is associated with an increased risk of complications, including severe microvascular disease, and that even a small amount of preserved β-cell secretory function prevents secondary complications.^8^ A recent international consensus on LADA management highlighted the need for large-scale clinical trials and prospective intervention studies and their paucity.^9^ Recent studies of type 1 diabetes have sought to treat the disease at an earlier stage, so-called stage 2, or to treat cases with therapeutic adjunct to insulin including using both immune therapy and a β-cell supportive agent, e.g., with an incretin effect.^10^

One such incretin agonist is a dipeptidyl peptidase 4 (DPP-4) inhibitor, such as saxagliptin, first approved by the US Food and Drug Administration, and now widely used in over 66 countries including China as an oral hypoglycaemia agent for type 2 diabetes. In a preliminary study, we showed the potential benefit of a DPP-4 inhibitor as an adjunct to insulin therapy.^11^ Moreover, previous studies based of the non-obese diabetes (NOD) mouse models suggested that DPP-4 inhibitors could limit insulitis, improve survival times of islet graft through reducing autoimmunity, and have beneficial effects on T cells with a transmembrane glycoprotein with DPP-4 enzymatic activity.^12,13^ In addition, vitamin D may be protective given its multiple regulatory roles in both metabolic and immune networks relevant to type 1 diabetes notably through key transcriptional effects of vitamin D receptor on β-cell survival.^14–16^ Dietary regular vitamin D may prevent diabetes in NOD mice and even potentially preserve endogenous β-cell function in patients with LADA.^17,18^

To this end, we conducted a multi-centre, randomized, controlled trial with large sample size sufficiently powered to examine whether a DPP-4 inhibitor (saxagliptin) with or without vitamin D as an adjunct to conventional therapy would preserve β-cell function and enhance clinical measures in patients with adult-onset autoimmune type 1 diabetes.

## Results

### Participants

Between Sep 2, 2015, and March 4, 2019, 386 individuals were assessed for eligibility, of whom 305 were randomly assigned to the saxagliptin group (*n* = 101), the saxagliptin plus vitamin D group (*n* = 103) or the conventional therapy group (*n* = 101; Supplementary Fig. 1). As prespecified, four randomized participants who did not meet the major inclusion criteria were excluded. Thus, 301 participants (*n* = 100, 102 and 99 in the saxagliptin, the saxagliptin plus vitamin D and the conventional therapy group, respectively) were included in full analysis set (FAS) following intention-to-treat principle. The 296 participants who received treatment and had at least one safety assessment were included in the safety set (Supplementary Fig. 1). Clinical and demographic characteristics were similar across three groups at baseline (Table 1). With complete adherence defined as 100% according to the time-weighted sum of percentage of compliance at each visit, the mean overall adherence to saxagliptin and vitamin D were 96.9 ± 5.0% and 94.5 ± 12.1%, respectively in active treatment groups. The mean levels of serum 25(OH)D were no different at baseline between the three groups, and were significantly higher in the saxagliptin plus vitamin D group than those in the saxagliptin group and the conventional therapy group at month 1 to month 24 (24.7 ng/mL,15.6 ng/mL and 17.8 ng/mL at month 24, respectively, *P* < 0.001) (Supplementary Fig. 2).

**Table 1:** Baseline Characteristics of the Participants

	Saxagliptin (*N* = 100)		Saxagliptin plus vitamin D (*N* = 102)	Conventional therapy (*N* = 99)
Age, year	43.3 ± 10.9		43.0 ± 13.6	43.0 ± 12.2
Female sex, no. (%)	44 (44.0)		38 (37.3)	42 (42.4)
Duration of diabetes, year	1.1 ± 0.9		0.9 ± 0.8	0.9 ± 0.7
BMI (kg/m^2^)	22.8 ± 3.7		22.9 ± 3.1	22.8 ± 4.2
BMI ≥ 25 kg/m^2^, no. (%)	21 (21.2)		24 (23.5)	25 (25.5)
Fasting plasma glucose, mmol/L	7.2 ± 2.7		7.4 ± 3.1	7.3 ± 2.7
Glycated haemoglobin, %	7.4 ± 1.8		7.8 ± 2.1	7.4 ± 1.8
Fasting C-peptide, pmol/L	243 (160-457)		282 (198-456)	267 (170-427)
2-h C-peptide AUC, pmol/L	1073 (560-1953)		1206 (698-1935)	1083 (652-2076)
Insulin use, n (%)	63 (63.0)		65 (63.7)	60 (61.2)
Daily insulin dose, U/kg/day^*^	0.37 ± 0.19		0.35 ± 0.18	0.40 ± 0.23
Serum 25(OH) D, ng/mL	19.7 ± 9.2		19.4 ± 8.0	20.4 ± 8.5
Distribution, *n* (%)				
<20.0	57 (57.0)		60 (58.8)	51 (52.0)
20.0–29.9	30 (30.0)		32 (31.4)	32 (32.7)
≥30.0	13 (13.0)		10 (9.8)	15 (15.3)
GADA, U/mL	372 (68-980)		322 (68-930)	279 (99-856)
HLA haplotypes presence (DR3/DR4/DR9), n (%)				
Absent		24 (25.0)	24 (24.5)	28 (30.1)
Present		72 (75.0)	74 (75.5)	65 (69.9)
Vitamin D receptor genotype, n (%)				
* Apa*I				
CC		48 (48.5)	49 (49.0)	49 (51.6)
CA + AA		51 (51.5)	51 (51.0)	46 (48.4)
* Taq*I				
AA		85 (85.0)	90 (89.1)	83 (86.5)
AG + GG		15 (15.0)	11 (10.9)	13 (13.5)
* Fok*I				
GG		30 (30.0)	31 (30.7)	23 (24.2)
AG + AA		70 (70.0)	70 (69.3)	72 (75.8)
* Bsm*I				
CC		90 (90.0)	93 (93.0)	89 (92.7)
CT + TT		10 (10.0)	7 (7.0)	7 (7.3)

Data are means ± SD, *n* (%) or medians (IQR). ^*^Among participants who use insulin at randomization. *BMI* body mass index, *AUC* area under the curve, *GADA* glutamic acid decarboxylase antibody, *HLA* human leukocyte antigen

### Efficacy

**Fig. 1:**
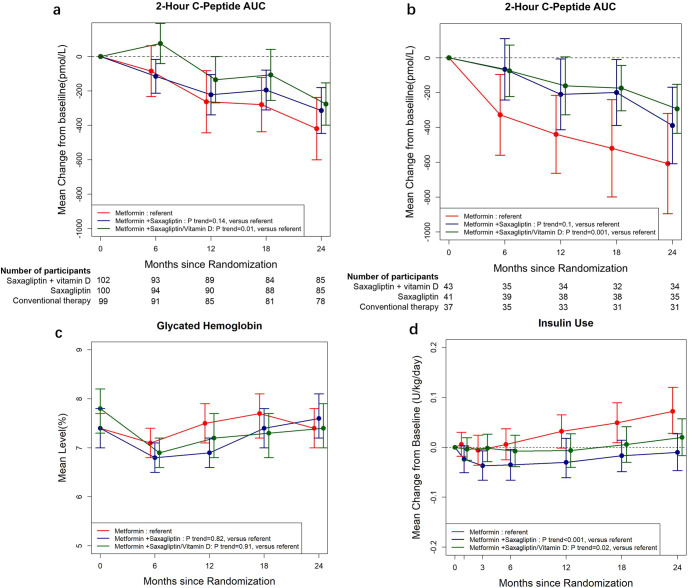
Effects of Saxagliptin and Saxagliptin plus Vitamin D on Secondary Endpoints. **a** Mean change from baseline through 24 months in the mixed-meal-stimulated C-peptide AUC among all participants in the saxagliptin, saxagliptin plus vitamin D and conventional therapy groups. **b** Mean change from baseline through 24 months in the mixed-meal-stimulated C-peptide AUC among participants with a high baseline GADA level of >577 U/mL in the saxagliptin, saxagliptin plus vitamin D and conventional therapy groups. **c** Mean glycated haemoglobin concentrations from baseline through 24 months. **d** Mean change from baseline through 24 months in total daily insulin use. Error bars indicate 95% confidence intervals; the mean values and their corresponding 95% confidence intervals are according to observed data. P for trend is obtained using mixed models for repeated measures. AUC area under the curve; GADA glutamic acid decarboxylase antibody

The primary endpoint was not reached. The change from baseline to 24 months in the fasting C-peptide was not significantly different in the saxagliptin plus vitamin D group (*P* = 0.18) and the saxagliptin group (*P* = 0.26) versus the conventional therapy group (Supplementary Fig. 3). However, the mean change from baseline to 24 months in the mixed-meal tolerance test (MMTT)-stimulated 2-h C-peptide AUC was -276 pmol/L (95% CI: -399, -153) in the saxagliptin plus vitamin D group, -314 pmol/L (95% CI: -447, -181) in the saxagliptin group, and -419 pmol/L (95% CI: -601, -238) in the conventional therapy group, which reflected 34.1% and 25.1% treatment effect (the ratio of mean change difference between treatment and conventional therapy group to the mean change value in the conventional therapy group), respectively. The mixed model for repeated measures indicated a significant difference between the group and time mean changes for participants receiving saxagliptin plus vitamin D compared with those in the conventional therapy group (*P* = 0.01; Fig. 1a). The participants in saxagliptin alone showed a non-significant smaller decrease of 2-h C-peptide AUC compared with conventional therapy (*P* = 0.14). The full least-squares models showed that there was no significant difference at 6 months, 12 months or 18 months (*P* > 0.05), and the mean change of 2-h C-peptide AUC was significantly different between the saxagliptin plus vitamin D group and the conventional therapy group at 24 months (*P* = 0.049, Supplementary Table 1). In addition, the proportion of participants who had a ΔC-peptide response at month 24 was 57.6% (49 of 85) in the saxagliptin plus vitamin D group, which was significantly higher than that in the conventional therapy group (37.2%, 29 of 78), for a between-group difference of 20.5% (95% CI, 4.2, 37.6, *P* = 0.02). The proportion of participants with ΔC-peptide response was only numerically higher in the saxagliptin group (51.8% [44 of 85]) as compared with the conventional therapy group (*P* = 0.10; Supplementary Fig. 4).

In the prespecified analyses stratified by glutamic acid decarboxylase antibody (GADA) levels, we found that the treatment effect was greater for participants with higher GADA levels at baseline (Fig. 1b and Supplementary Fig. 5). Notably, for participants with a baseline GADA level of 577 U/mL or higher (60^th^ percentile cut-off point), the mean change from baseline to 24 months in the 2-h C-peptide AUC was -293 pmol/L (95% CI: -434, -152) in the saxagliptin plus vitamin D group, and -389 pmol/L (95% CI: -608, -169) in the saxagliptin group and -607 pmol/L (95% CI: -895, -320) in the conventional therapy group, reflecting a treatment effect of 51.7% and 35.9%, respectively (Fig. 1b). The repeated measures of ANOVA revealed the saxagliptin plus vitamin D group was significantly different compared with the conventional therapy group (*P* = 0.001), and the full least-squares models indicated a significant difference of mean change of 2-h C-peptide AUC between the saxagliptin plus vitamin D group and the conventional therapy group at 12 months, 18 months and 24 months (*P* = 0.023, 0.038 and 0.013, respectively), and a borderline significant difference at 6 months (*P* = 0.06). Similar results were observed for participants with a baseline GADA level over 50^th^, 70^th^, 75^th^ or 80^th^ percentile cut-off points (Supplementary Table 1).

### Other secondary endpoints

To assess glycemic control, we measured glycated haemoglobin (HbA_1C_) concentrations at baseline, 6 months, 12 months, 18 months and 24 months. Average HbA_1C_ concentrations remained similar among the saxagliptin plus vitamin D, the saxagliptin and the conventional therapy group (baseline: 7.8%, 7.4% and 7.4%, respectively; 24 months: 7.4%, 7.6% and 7.4%, respectively). The repeated measures of mixed models did not reveal a significant difference in change of the HbA_1C_ levels from baseline to 24 months across groups (all *P* ≥ 0.82; Fig. 1c and Supplementary Table 2).

To examine the usage of exogenous insulin in order to maintain glycemic control, we conducted analyses to evaluate the change of total daily insulin use. At baseline, total daily insulin use was 0.25, 0.24 and 0.23 U/kg/day for the saxagliptin plus vitamin D, the saxagliptin and the conventional therapy group, respectively. The total daily insulin use increased 30% in the conventional therapy group that was concordant with the disease progression, whereas the increases were only 4.2% in both saxagliptin plus vitamin D and saxagliptin alone groups (*P* = 0.02 and 0.0002, respectively; Fig. 1d and Supplementary Table 3).

### Subgroup analysis

**Fig. 2:**
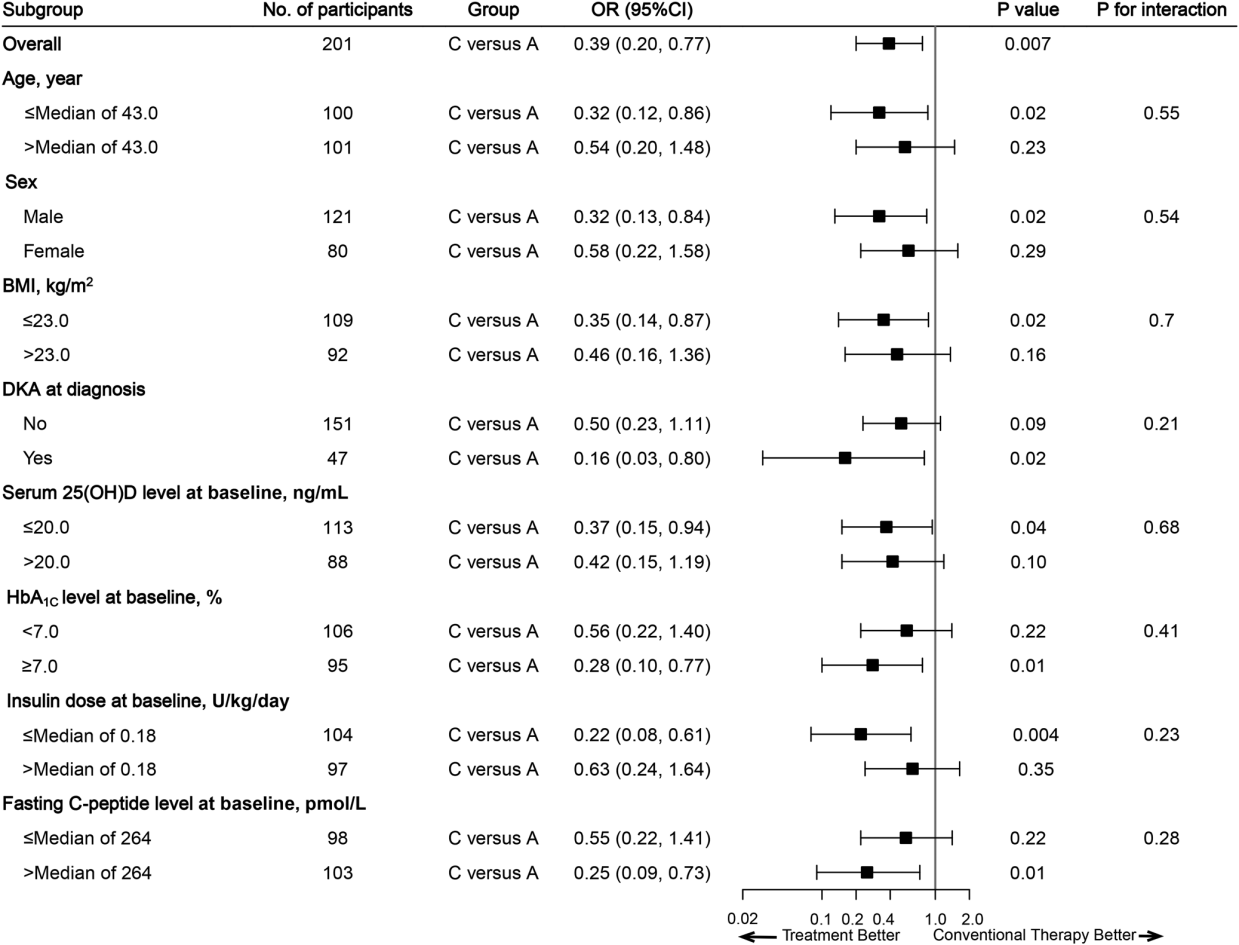
Subgroup Analysis of Responses to Saxagliptin plus Vitamin D. The forest plot presents the odds ratios and their 95% confidence intervals for the changes from baseline through 24 months in the C-peptide AUC (two categories: low versus high, median cut-off point) in the saxagliptin plus vitamin D group as compared with the conventional therapy group, stratified by baseline factors (two categories of each). The logistic regression model was adjusted for age at recruitment, sex, baseline C-peptide (log[AUC + 1]), baseline vitamin D concentration and DKA at diagnosis. Group A: conventional therapy, Group C: saxagliptin plus vitamin D; AUC area under the curve, BMI body mass index, DKA diabetic ketoacidosis

Results from logistic regression models revealed that participants receiving saxagliptin plus vitamin D was associated with a 61% reduced risk of significant decrease in the 2-h C-peptide AUC response at 24 months when compared with those in the conventional therapy group (OR = 0.39, 95% CI: 0.20, 0.77; *P* = 0.007, Fig. 2), while the association was not significant for participants receiving saxagliptin as compared with those receiving conventional therapy (OR = 0.57, 95% CI: 0.30, 1.11; *P* = 0.10). In the predefined subgroup analyses, we assessed effect modification of the treatment-2-h-C-peptide AUC (mean change at 24 months) by baseline factors, and the results were generally similar across all pre-selected factors, including age at recruitment, sex, baseline values of BMI, serum 25(OH)D level, HbA_1C_ level, insulin treatment, fasting C-peptide, diabetic ketoacidosis (DKA), as well as HLA subtype (HLA-DR3, -DR4 or -DR9), number of alleles of HLA-DR3, -DR4 or -DR9, and vitamin D receptor (VDR) gene (*Apa*I, *Fok*I, and *Bsm*I). The treatment effect was, however, modified by VDR gene of *TaqI* (*P interaction* = 0.018; Supplementary Fig. 6). The response to saxagliptin plus vitamin D as compared with conventional therapy was larger among participants with the AG genotype in the VDR gene of *TaqI* than those with the AA genotype, although there were only 24 participants with the AG genotype of *TaqI* (OR = 0.01 [95% CI: 0, 0.35]).

### Safety

Overall, saxagliptin and vitamin D were well tolerated. The incidence of at least one of any adverse events during the 2-year follow-up was 77 (77.8%), 72 (72.7%) and 78 (79.6%) in the saxagliptin group, saxagliptin plus vitamin D group and conventional therapy group, respectively (Table 2). Adverse events of interest, including infection, renal stones, hypoglycemia, DKA and hypercalcemia, were not significantly different across all groups. No participants receiving vitamin D developed hypervitaminosis D. Hypoglycaemia events were reported in 41 (41.4%), 39 (39.4%), and 35 (35.7%) in the saxagliptin group, saxagliptin plus vitamin D group and conventional therapy group, respectively. One hypoglycaemia coma which was resolved after emergency management was reported in a patient in the saxagliptin group during trial, who was also administrated with multiple insulin injection. DKA was infrequent (two participants in the saxagliptin group, three in the saxagliptin plus vitamin D group and one in the conventional therapy group). A total of 30 serious adverse events were reported and were distributed equally across all groups, of which none was identified as probably or definitely related to trial agent.

**Table 2:** Adverse Events (Safety Analysis Population)

Adverse Events	Saxagliptin (n = 99)		Saxagliptin plus vitamin D (n = 99)		Conventional therapy (n = 98)	
	Participants (n, %)	Events (n)	Participants (n, %)	Events (n)	Participants (n, %)	Events (n)
Any adverse events	77 (77.8)	201	72 (72.7)	179	78 (79.6)	208
Leading to discontinuation	2 (2.0)	2	5 (5.1)	5	4 (4.1)	4
Related to trial agent^*^	5 (5.1)	5	6 (6.1)	6	6 (6.1)	9
Any serious adverse events	6 (6.1)	6	10 (10.1)	12	11 (11.2)	12
Anorexia, nausea, or vomiting	3 (3.0)	3	0	0	1 (1.0)	1
Renal stones	21 (21.2)	24	18 (18.2)	20	24 (24.5)	28
Any infection	7 (7.1)	12	12 (12.1)	21	10 (10.2)	13
Increased hepatic enzyme	7 (7.1)	9	7 (7.1)	7	4 (4.1)	4
Diarrhoea	5 (5.1)	7	1 (1.0)	1	3 (3.1)	3
Skin disorders	5 (5.1)	6	1 (1.0)	1	1 (1.0)	1
Headache	1 (1.0)	1	0	0	1 (1.0)	2
Allergy	0	0	1 (1.0)	1	1 (1.0)	1
Cancer	0	0	1 (1.0)	1	1 (1.0)	1
Death	0	0	0	0	0	0
Hypoglycemia^†^	41 (41.4)	213	39 (39.4)	214	35 (35.7)	199
Severe hypoglycemia^†^	16 (16.2)	28	12 (12.1)	28	15 (15.3)	35
Diabetic ketoacidosis	2 (2.0)	2	3 (3.0)	3	1 (1.0)	1
Other monitored safety conditions^‡^						
Lymphocyte count decreased	6 (6.1)	8	3 (3.0)	8	2 (2.0)	3
Hypercalcemia	1 (1.0)	1	1 (1.0)	1	1 (1.0)	1
Serum 25(OH)D > 100 ng/mL	0	0	0	0	0	0

^*^Events related to trial agents were defined as possibly, probably, or definitely related to the trial agent by trial-site investigators

^†^Hypoglycaemia data were considered separately. Hypoglycemia was defined as a confirmed blood glucose <3.9 mmol/L irrespective of clinical symptoms or have symptoms consistent with hypoglycemia and prompt recovery after oral carbohydrate, intravenous administration of glucose or glucagon with or without glucose measurement. Severe hypoglycaemia was defined as a blood glucose <3.0 mmol/L or a hypoglycaemia event resulting in altered mental and physical status

^‡^Lymphocyte count decreased was defined as a lymphocyte count lower than 0.8x10e9. Hypercalcemia was defined as two consecutive measurements of an uncorrected serum calcium level higher than the upper limit of the normal range for the clinical laboratory at each trial site

## Discussion

In this randomized controlled trial with 24-month follow-up, saxagliptin and vitamin D when added to the conventional regimen including metformin and insulin, resulted in improved endogenous pancreatic β-cell function, as indicated by the 2-h C-peptide AUC and decreased exogenous insulin requirement. The protective effect of saxagliptin and vitamin D was most prominent among patients with higher GADA level, who usually have a more rapid decline in β-cell function. This study provides the first clear evidence that an adjunctive combination of saxagliptin and vitamin D to insulin and metformin can be valuable in the management of adult-onset type 1 diabetes. Given the paucity of data in this field, and the uncertainty regarding optimal therapy, our data provide evidence of the beneficial effects of even modest preservation of C-peptide for saxagliptin combined with vitamin D, and deserves further study.^9,19,20^ Additionally, this large trial validates our 1-year pilot trial in a subgroup of patients from three centres, which identified potential effects of saxagliptin combined with vitamin D in β-cell preservation.^21^

Although stimulated C-peptide levels have been considered as a gold standard of β-cell function evaluation in type 1 diabetes trials,^22,23^ we chose fasting C-peptide as the primary endpoint based on its easy practical procedure reducing demands on patients, the high correlation with simulated C-peptide and our previous trial of sitagliptin in patients with LADA.^11^ Fasting C-peptide levels were also chosen as the primary endpoints in the trials of GAD65 antigen therapy.^24,25^ However, MMTT-stimulated C-peptide provide more complete data on endogenous insulin secretion, thereby has a better discrimination of difference between groups than fasting C-peptide. Although saxagliptin and vitamin D did not affect fasting C-peptide at month 24, the secondary endpoint based on 2-h C-peptide AUC was met for saxagliptin with vitamin D treatment, supporting the notion that saxagliptin and vitamin D preserve residual C-peptide.

This current study showed that saxagliptin alone led to a non-significant C-peptide preservation compared with conventional therapy. Findings from previous small trials of DPP-4 inhibitors in LADA have been conflicting,^11,26–29^ the effect varying substantially due to factors including the source population, sample size, specific time window for intervention and study design. Our data supports a previous post-hoc analysis of pooled type 2 diabetes trials that saxagliptin monotherapy led to a non-significant trend towards C-peptide preservation in LADA patients compared with those receiving placebo.^28^ Further, compared with our previous trial of sitagliptin which showed a beneficial effect,^11^ the present trial included patients with relatively lower β-cell function (i.e. fasting C-peptide 100-200 pmol/L), in whom any agent may be less efficacious because of a paucity of residual β cells to be preserved.^1^ Our results are consistent with the proposal from recent reviews that cases with a random serum C-peptide less than 200 or 300 pmol/L should be treated with insulin as for insulin-dependent type 1 diabetes.^2,3,9^ Another possible reason for null results with saxagliptin is that the effect was milder than the anticipated effect, and a larger sample size are warranted to assess the efficacy of saxagliptin as monotherapy. DPP-4 inhibitors block the breakdown of glucagon-like peptide 1 (GLP-1). However, trials of liraglutide^10^ and albiglutide^30^ found that these GLP-1 receptor agonists alone had no effect on β-cell preservation, suggesting that the effect of saxagliptin may be mediated in a GLP-1 independent way. As expected, we also found that participants with saxagliptin had a significantly lower insulin dose than those with conventional therapy, which could result from the glycemic effect of saxagliptin and the potential mild effect on β-cell preservation.

Our findings that adjunctive vitamin D to saxagliptin monotherapy seems to enhance treatment effect indicated a valuable effect of vitamin D on C-peptide preservation. Due to the lack of vitamin D monotherapy arm, the contribution of each component in the combination therapy is unclear. But given that saxagliptin alone showed a non-significant trend of benefits towards C-peptide preservation, we speculate that both vitamin D and saxagliptin might contribute to the efficacy of combination therapy. The effect of vitamin D as monotherapy on C-peptide is not clear. Pre-clinical studies showed a pleiotropic role in islet autoimmunity. Vitamin D supplementation prevented diabetes accompanied with reduced inflammatory CD8 + T cells and increases regulatory T cells in NOD mice,^18^ and reduced β-cell failure under stressors and inflammation through a vitamin D receptor signalling in β cells.^15^ In contrast, most trials of vitamin D in type 1 diabetes had negative results,^31,32^ although our previous small trial of active vitamin D analogue showed the effect of β-cell preservation in patients with LADA.^17^ The mild effects of saxagliptin and vitamin D in the present and the past studies indicated that these two agents may have synergistic mechanism in immune modulation. Future factorial designed trials and animal studies are warranted to explore the efficacy of vitamin D in combination strategy or as monotherapy and the underlying synergistic mechanism.

Autoimmune diabetes is highly heterogeneous in clinical features and autoimmunity.^33,34^ For example, high GADA level identifies an endotype of LADA patients with a higher autoimmune response and a more rapid decline in β-cell function,^35^ suggesting therapies with immunomodulation might be more likely to have a beneficial effect, potentially explaining why a greater treatment response was noted in patients with high GADA level. Moreover, given the faster β-cell deterioration in patients with high GADA level, the between-group difference could be easier to observe in such high GADA level patients than in those with lower GADA level; perhaps, a longer follow-up might be required to delineate beneficial effects. This present finding illustrated the potential for clinicians to target adult-onset autoimmune diabetes patients in a precision medicine approach to their management.^2,3^

The strengths of this study include its rational design of combination therapy, large sample size, completeness of long-term follow-up for clinical examinations and MMTT tests, as well as centralized repeated measurements of C-peptide, 25(OH)D and GADA. Moreover, the overall adherence was high, and the use of outside-of-trial vitamin D supplements was similarly low across three groups (two, five and six participants in the saxagliptin plus vitamin D group, the saxagliptin alone group and in the conventional therapy received additional vitamin D3 or calcitriol, respectively). The large sample size enabled evaluation with substantial statistical power for treatment efficacy, even within a range of study subgroups. Importantly, saxagliptin did not lead to an inferior response here compared with conventional treatment. Given that, insulin aside, there is no other therapy approved for such cases of autoimmune diabetes, our trial suggests oral saxagliptin and vitamin D should be considered.

Several limitations in this study should be mentioned. Firstly, we did not include an arm of vitamin D monotherapy given the difficulty in recruitment, so we are unable to make any conclusions about the efficacy of vitamin D and the contribution of each individual component of the combination therapy. Secondly, the homogenous population of Asian participants in this study may limit generalizability of these findings to other ethnic groups. Our findings should be re-examined in other populations of different ethnic/racial participants. Third, it is recognised that a proportion of cases with autoantibody positive adult-onset diabetes cases may have a false positive autoantibody test, for example up to 20% of cases in our study might have type 2 diabetes with a false positive GADA. However, our results remained unchanged after excluding patients with transient positive GADA. Two observations further suggest that false positivity is not relevant to our broad conclusion. First, the optimum response to saxagliptin and vitamin D was in those with the highest, not lowest, level of GADA. Second, our cases largely had C-peptide in the category designated type 1 diabetes or ‘uncertain classification’ in the recent reports.^3^ However, those reviews also suggested that cases with autoantibody positivity but with random C-peptide >600–700 pmol/L should be treated as though they have type 2 diabetes.^2,3,9^ Since oral saxagliptin is approved therapy for type 2 diabetes and since we now show that cases with adult-onset type 1 diabetes also have a beneficial effect from oral saxagliptin and vitamin D added to conventional therapy, it implies that this initial therapeutic strategy, if confirmed, can be used without a need to initially circumvent precise classification of the type of diabetes. Fourth, owing to the absence of placebo control and non-double-blind treatment, we cannot rule out the risk of biased supplemental care, as with other open-labelled trials. Fifth, though saxagliptin and vitamin D was well-tolerated in 2-year follow-up, potential chronic and infrequent complications may require longer observation and larger sample size.

In conclusion, oral saxagliptin and vitamin D added to conventional therapy regimen ameliorated pancreatic β-cell function loss in patients with adult-onset type 1 diabetes, especially those with high GADA levels. This large-scale trial demonstrated an effective and novel approach to broaden current treatment strategies in cases with adult-onset type 1 diabetes, including LADA, and provides additional evidence for precision medicine in the management of this disease.

## Materials and methods

### Study design

The ADVENT (Adding DPP-4 inhibitor and vitamin D for adult-onset autoimmune diabetes) study is a prospective, multi-centre, open-label, randomized controlled trial, comprising a 6-week run-in period and then 24-month treatment period. The trial was conducted from September 2015 to December 2020 at 36 sites across 18 provinces and 3 province-level municipalities in China in accordance with the principles of the Declaration of Helsinki, and the International Conference on Harmonisation guidelines for Good Clinical Practice. Ethical committee approval was obtained at the Ethics Committee of the Second Xiangya Hospital, Central South University and each participating site. All participants provided signed, written informed consent before trial entry.

### Participants and randomization

The participants between 18 and 70 years of age who received a diagnosis of diabetes according to 1999 World Health Organization (WHO) criteria at 18 years or older within 4 years, and were initially positive for GADA, had a fasting C-peptide level of at least 100 pmol/L or 2-h postprandial C-peptide level of at least 200 pmol/L after MMTT, were eligible for participation in the trial. Participants who received DPP-4 inhibitors, GLP-1 analogues, GLP-1 receptor agonists and thiazolidinediones within 8 weeks prior to randomization and had a medical history of other clinically significant diseases were excluded.

Participants were randomly assigned 1:1:1 by a central randomization system to receive metformin with or without insulin (conventional therapy), saxagliptin alone or saxagliptin plus vitamin D as add-on drugs to conventional therapy. Randomization was stratified by the fasting C-peptide (<300 pmol/L or ≥300 pmol/L), GADA level (<180 or ≥180 U/mL)^36^ and BMI (<25 or ≥25 kg/m^2^).

### Procedures

Participants in the saxagliptin group took 5-mg of saxagliptin once daily. Participants in the saxagliptin plus vitamin D group received saxagliptin in the same manner concomitantly with 2000U of vitamin D3 orally once daily. The dose of metformin was individualized by investigators to achieve optimal glycemic control. In case of inadequate glycemic control, any insulin regimen was approved at the investigator’s discretion. No additional antidiabetic treatment was allowed during the trial. Participants were required to record insulin dose, concomitant use of drug, self-monitoring blood glucose levels and hypoglycaemic events in participant diaries at home. Unused investigational drug was returned at each visit to assess adherence.

Follow-up clinic visits after randomization were scheduled for month 1, 2, 3, 6, 10, 12, 18, 24 with telephone visits at every-4-week interval between the clinic visits. MMTTs contains 1658kJ (72% from carbohydrate, 9.5% from protein, 18.5% from fat) and were scheduled at baseline and month 6, 12, 18 and 24 to evaluate β-cell function. Saxagliptin was held for 72 h before the MMTT, and insulin injection was withheld on the day of the MMTT. Fasting C-peptide was taken after overnight fasting for at least 8 h. (Supplementary Methods). C-peptide and serum 25(OH)D levels were assessed with a chemiluminescence method using the Adiva Centaur System kit (Siemens, Munich, Germany). GADA was assessed using radioligand assay, with sensitivity and specificity being 82% and 97.8% confirmed by Islet Autoantibody Standardization Program (IASP). C-peptide, 25(OH)D and islet autoantibodies were measured by the core laboratory of the Second Xiangya Hospital, Central South University (Changsha, China) (Supplementary Methods).

### Outcomes

The primary endpoint was β-cell function measured via the absolute change from baseline to month 24 in fasting C-peptide levels. Prespecified secondary endpoints were the change from baseline to month 6, 12, 18 in fasting C-peptide levels, the change from baseline to month 24 in the area under the concentration-time curve for MMTT-stimulated C-peptide levels over 2 h (2-h C-peptide AUC) and the proportion of participants who had a ΔC-peptide response at month 24 (defined as either having an increase in ΔC-peptide levels versus baseline or not decreasing >40%). Other prespecified endpoints were HbA_1C_ levels, insulin dose (based on 7 days before clinical visit).

Safety measures included adverse events, hypoglycaemia and hypercalcemia reported by patients or noted by the investigator. Vital signs, physical examination and clinical laboratory tests were assessed at each clinic visits. Renal ultrasound was conducted at baseline and month 1, 3, 6, 12, 18, 24. Hypoglycaemia was defined as a confirmed blood glucose <3.9 mmol/L irrespective of clinical symptoms or have symptoms consistent with hypoglycaemia and prompt recovery after oral carbohydrate, intravenous administration of glucose or glucagon with or without glucose measurement. Hypercalcemia was defined as two consecutive measurements of an uncorrected serum calcium level higher than the upper limit of the normal range for the clinical laboratory at each trial site.

### Statistical analysis

By assuming changes in the mean value of fasting C-peptide from 24-month to baseline in the conventional therapy group to be -150 pmol/L (SD = 85), and in the treatment group to be -108 pmol/L (SD = 53), and on the basis of randomization allocated in a 1:1:1 ratio, and a two-sided t test with the type I error rate of 0.025 in either treatment groups compared with conventional therapy (overall alpha of 0.05 for both tests), a sample size of 95 participants would provide 90% power to detect a statistically significant treatment difference, considering a drop-out rate of 20%. Thus, 100 participants for each group were randomly assigned.

The endpoint analyses were based on the prespecified FAS population following intention-to-treat principle, defined as those who received at least one dose of treatment. We computed 2-h C-peptide AUC based on the trapezoidal method with the timepoints (0-, 60- and 120-min) measurements of C-peptide during the MMTT, and we conducted a logarithmic transformation on the fasting C-peptide (logFCP) and C-peptide AUC data (log[AUC + 1]) to obtain the normal distribution. ΔC-peptide was computed by C-peptide at 120 min during MMTT minus fasting C-peptide. The primary endpoints compared the absolute difference from 24 months to baseline in the fasting C-peptide between two treatment groups and the conventional therapy group were based on a the mixed model for repeated measures adjusting for age at recruitment, sex, time, treatment-by-time interaction, baseline C-peptide (log[AUC + 1]), baseline C-peptide-by-time interaction, baseline vitamin D concentration, insulin or metformin dosage and DKA at diagnosis. In secondary endpoint analysis, the rate of mean change of C-peptide AUC from baseline to 6, 12, 18 and 24 months was estimated by the mixed model for repeated measures, which included the fixed, categorical effects of group, time, treatment-by-time interaction, sex and the presence of (DKA at diagnosis, as well as the fixed, continuous covariates of baseline C-peptide (log[AUC + 1]), baseline C-peptide-by-time interaction, age at recruitment and baseline serum 25(OH)D concentration. An unstructured covariance was used to model the within-patient errors. Based on the relationship between levels of GADA and the severity of islet autoimmunity and endogenous β-cell function, prespecified treatment effect analyses using mixed effects models were further stratified by GADA levels at baseline (predefined cut-offs: ≤180, 180-≤577 [60^th^ percentile] and >577 U/mL; post-hoc cut-offs: 180 -≤378[50^th^ percentile], >378, 831, 887 and 955 U/mL [50^th^, 70^th^, 75^th^, 80^th^ percentile, respectively]). We used the similar approach of mixed effects models to examine the treatment effect by comparing the changes from baseline to 24 months in the glycated haemoglobin levels and insulin usage. We used a logistic-regression model adjusting for age at recruitment, sex and baseline levels to compare ΔC-peptide response between groups.

In the prespecified subgroup (exploratory) analyses, we used logistic regression models to examine odds ratios (ORs) and their 95% confidence intervals (CIs), for the association between the changes from baseline through 24 months in the C-peptide AUC (two categories: low versus high, median cut-off point) and treatment group, adjusted for age at recruitment, sex, baseline C-peptide (log[AUC + 1]), baseline vitamin D concentration and DKA at diagnosis. Stratification analyses were performed based on age at recruitment, sex, baseline values of BM, DKA, serum 25(OH)D level, HbA1C level, insulin treatment, fasting C-peptide, as well as HLA subtype, number of haplotypes of HLA-DR3, -DR4 or -DR9, and vitamin D receptor (VDR) gene *(Apa*I, *Fok*I, *Taq*I and *Bsm*I). We tested if the examined associations differed significantly according to these prespecified factors by including a cross-product term between the factor and treatment group in the logistic regression.

All analyses were conducted using SAS software (version 9.4, SAS Institute Inc.) and R statistical software (version 3.5.2, Vienna, Austria). All reported P values are two-sided, with the threshold of statistical significance of 0.05, and all reported confidence intervals are 95% confidence intervals. Missing data were assumed to be missing randomly.

## Supplementary information

Supplementary_Materials

Study Protocol

## Data Availability

Data collected for the study and presented herein will be made available to others. Individual participant data will be shared in datasets in a deidentified format. Additional documents including the study protocol, statistical analysis plan and informed consent form will be available. Data requests should be sent by email to the corresponding author (zhouzhiguang@csu.edu.cn), who will partner with the National Clinical Research Center for Metabolic Diseases (Changsha, China) to supply the requested information after approval of a proposal.
