# Supplementary material for: Combination therapy with saxagliptin and vitamin D for the preservation of β-cell function in adult-onset type 1 diabetes: a multi-center, randomized, controlled trial
Source: Signal Transduct Target Ther. 2023 Apr 20;8:158. doi: 10.1038/s41392-023-01369-9 (PMC10115841; doi:10.1038/s41392-023-01369-9)
Supplement: Supplementary file 1 — Supplementary_Materials [file 41392_2023_1369_MOESM1_ESM.docx]

Supplementary Materials for

Combination Therapy with Saxagliptin and Vitamin D for the Preservation of β-cell Function in Adult-onset Type 1 Diabetes: A Multi-center, Randomized, Controlled Trial

Xiang Yan, MD #; Xia Li, MD #; Bingwen Liu, MD #; Jiaqi Huang, PhD #; Yufei Xiang, MD ; Yuhang Hu, MD ; Xiaohan Tang, MD ; Ziwei Zhang, MD ; Gan Huang, MD ; Zhiguo Xie, PhD ; Houde Zhou, PhD ; Zhenqi Liu, MD ; Xiangbing Wang, MD ; Richard David Leslie, MD ; Zhiguang Zhou, MD on behalf of ADVENT Study Group.

# Those authors contributed equally.

Correspondence to: [zhouzhiguang@csu.edu.cn](mailto:zhouzhiguang@csu.edu.cn) and r.d.g.leslie@qmul.ac.uk

**This PDF file includes:**

Supplementary Methods

Supplementary Text

Figures. S1 to S6

Tables S1 to S3

Materials and Methods

Glutamic Acid Decarboxylase Antibody (GADA) Methods: GADA was detected by radioligand assays in duplicate in the central laboratory of the Second Xiangya Hospital, Central South University (Changsha, China), all patients with GADA positivity were repeated after one week. Based on the 99th percentile observed in 405 healthy participants, the cut-off value of positivity for GADA was 18 U/ml of WHO units. In the 2016 islet autoantibody standardization programme (IASP 2016), the sensitivity and specificity in our laboratory were 82% and 97.8% for GADA.

2-hour Mixed Meal Tolerance Tests (MMTTs) Methods: After overnight fasting, a standardized, commercially available cup noodle (Wugudaochang®) was consumed before 10 a.m., which contains 1658kJ (72% from carbohydrate, 9.5% from protein, 18.5% from fat). Saxagliptin was held for 72 hours before the MMTT, and insulin injection was withheld on the day of the MMTT. Samples were obtained for glucose and C-peptide at 0, 60, and 120 minutes. Blood sampling, storage and shipping were handled according to a standard operating procedure in the trial specific laboratory manual. C-peptide levels were measured in the central laboratory of the Second Xiangya Hospital, Central South University (Changsha, China).

Supplementary Text

Investigators List of ADVENT Study group

Linong Ji, Xiaoling Cai, Ling Chen, Peking University People’s Hospital; Lixin Guo, Qi Pan, Xiaofan Jia, Department of Endocrinology, Beijing Hospital, National Center of Gerontology, Institute of Geriatric Medicine, Chinese Academy of Sciences; Zhongyan Shan, Yanli Cao, Yaxin Lai, Zhuo Zhang, Huimin Hou, Hanbing Liu, The First Hospital of China Medical University; Hanqing Cai, Xiying Fu, Mo Li, Lili Ning, The Second Hospital of Jilin University; Yadong Sun, Yan Ma, Ying Xing, People’s Hospital of Jilin Province; Tao Yang, Mei Zhang, Hao Dai, The First Affiliated Hospital with Nanjing Medical University; Liyong Yang, Yan Sunjie, Peiwen Wu, Xiaofang Yan, The First Affiliated Hospital of Fujian Medical University; Fang Wang, Yangang Wang, Wenshan Lv, Qu Chen, Hong Chen, The Affiliated Hospital of Qingdao University; Qifu Li, Rong Li, The First Affiliated Hospital of Chongqing Medical University; Jing Liu, Limin, Tian, Yunfang Wang, Suhong Wei, Gansu Provincial Hospital; Gebo Wen, Jianghua Liu, Xinhua Xiao, Jianping Qin, The First Affiliated Hospital of University of South China; Jian Kuang, Jianhao Pei, Zhong Chen, Xiaoying Fu, Haixia Guan, Shuting Zhang, Shuiqing Lai, Department of Endocrinology, Guangdong Provincial People’s Hospital, Guangdong Academy of Medical Sciences, Guangzhou, China; Shaoda Lin, Kun Lin, Shaoyu Zheng, The First Affiliated Hospital of Shantou University Medical College; Xiaohong Niu, Yan Sun, Li Li, Heji Hospital Affiliated to Changzhi Medical College; Zhiguang Zhou, Xia Li, Houde Zhou, Gan Huang, Yufei Xiang, Xiang Yan, Chao Deng, Yanfei Wang, Yiyu Zhang, Yuhang Hu, Binwen Liu, Xiaohan Tang, The Second Xiangya Hospital of Central South University; Huibiao Quan, Leweihua Lin, Hainan General Hospital; Hongyu Kuang, Weihua Wu, Changwei Yang, Guanyiqing Jiang, The First Affiliated Hospital of Harbin Medical University; Yuling He, The First Affiliated Hospital of Guangxi Medical University; Xiaoyan Chen, Dongling Li, Yuyu Tan, The First Affiliated Hospital of Guangzhou Medical University; Ling He, Li Chen, Yijun Xie, Wen Zhou, Guangzhou First People’s Hospital; Chao Zheng, Chenwei Wu, The Second Affiliated Hospital of Wenzhou Medical University; Jianying Liu, Zhifang Yang, The First Affiliated Hospital of Nanchang University; Ling Hu, Ying Hu, Xia Sheng, The Third Affiliated Hospital of Nanchang University; Huijuan Yuan, Xuqing Li, Shasha Tang, Xiaoyang Shi, Henan Provincial People’s Hospital; Heng Su, Yang Ou, The First People’s Hospital of Yunnan Province; Jianping Wang, Shanshan Deng, Aihua Jiang, Chanjie Xia, The Second Affiliated Hospital, Hengyang Medical School, University of South China; Changqing Luo, Shan Liu, Kang Lei, Yueyang Central Hospital; Zhiming Deng, Shenglian Gan, Ting Sun, The First People’s Hospital of Changde City; Qiuxia Huang, Guangying Huang, Dongguan People’s Hospital; Yi Zhang, Zhenzhen Hong, Quanzhou First Hospital Affiliated to Fujian Medical University; Caifeng Yan, Ting Liu, Lu Tan, Northern Jiangsu People’s Hospital; Zhiwen Liu, Liming Wu, Mei Guo, Yanfei Jiang, Xulei Zheng, Shanghai Xuhui District Central Hospital; Meibiao Zhang, Ling Zhang, Jingjin Yang, Xiaoyu Lu, Jiaojiao Yang, The First People’s Hospital of Huaihua; Hongwei Jiang, Yujin Ma, Liujun Fu, Jie Liu, Liping Li, Wenbo Zhang, Hui Zhang, Xiaoli Liang, Zhen Zhang, Guixia Zhao, Fang Liu, The First Affiliated Hospital, and College of Clinical Medicine of Henan University of Science and Technology; Hui Fang, Jinli Tian, Xueling Sun, Tangshan Gongren Hospital; Hui Sun, The Affiliated Hospital of Inner Mongolia Medical University; Guoping Wang, Chunyan Bao, The Second Affiliated Hospital of Baotou Medical College; Rui Zhang, Qing Chang, The People’s Hospital of DaLaTe; Jia He, Liyan Hu, Naval Medical University.

Figure. S1. Screening, Randomization, and Follow-up

**
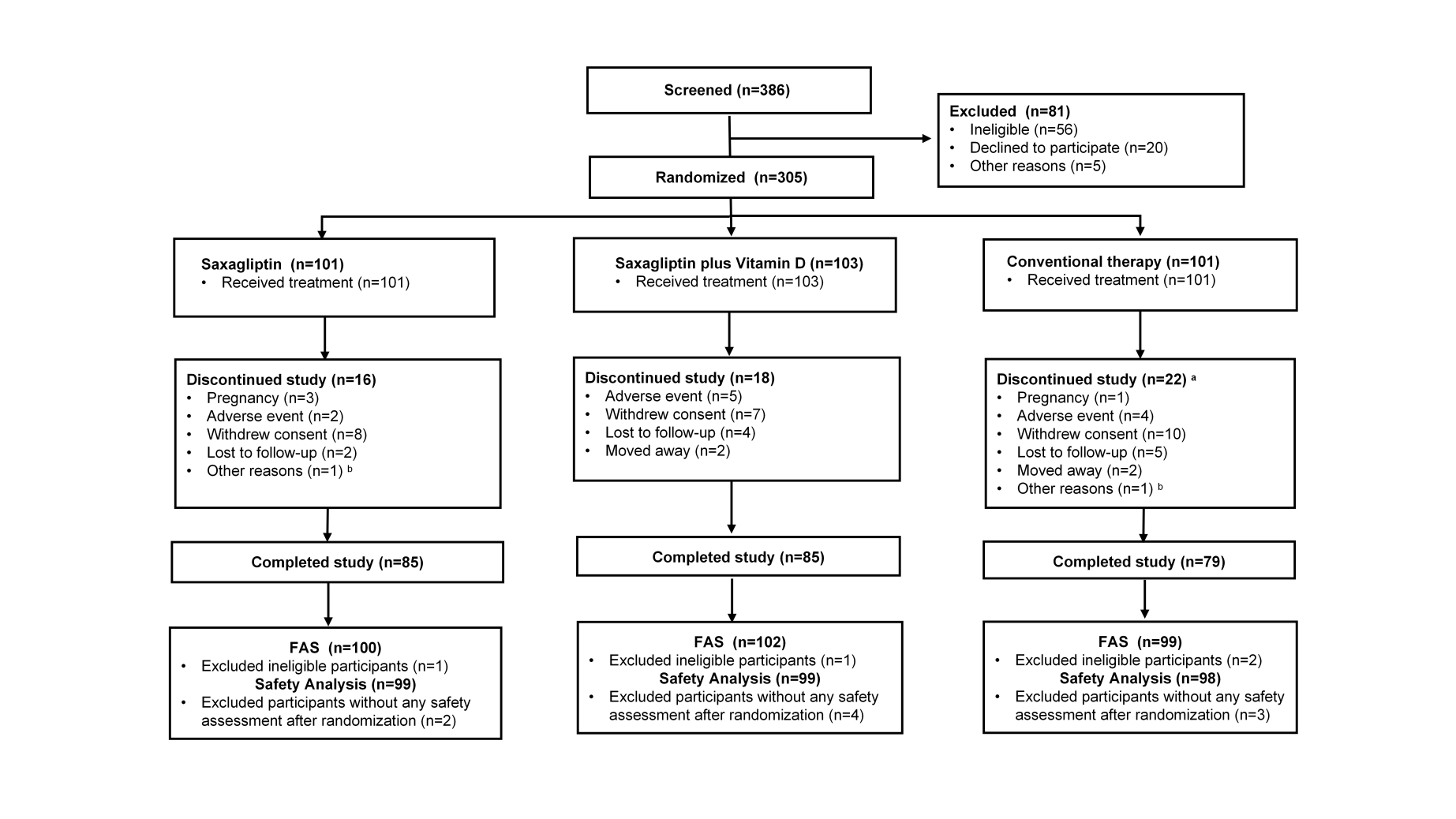
**

One ineligible participant with low C-peptide level in the saxagliptin group, one ineligible participant with age<18 years in the saxagliptin plus vitamin D group, and two ineligible participants with negative GADA and with low C-peptide level in the conventional therapy group were excluded from FAS according to the protocol. a. One participant fulfilled pregnancy and adverse event simultaneously. b. Randomization error. FAS=full analysis set.

Figure. S2. Serum 25(OH)D Levels during the Study

Bars represent standard deviation around mean.

Figure. S3. Effects of Saxagliptin and Saxagliptin plus Vitamin D on Fasting C-peptide

**
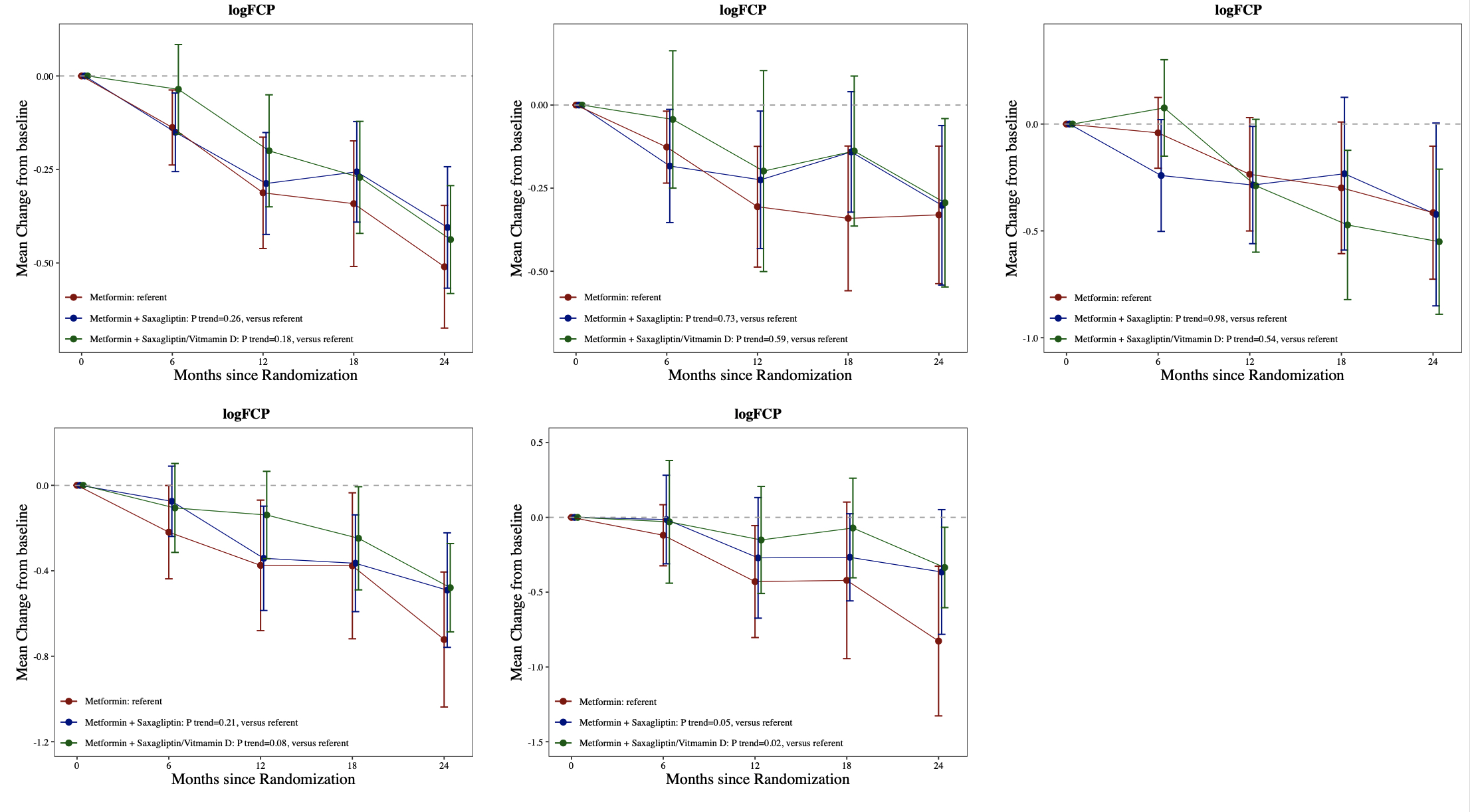
**

Error bars indicate 95% confidence intervals; the mean values and their corresponding 95% confidence intervals are according to observed data. P for trend is obtained using mixed models for repeated measures.

Figure. S4. Effects of Saxagliptin and Saxagliptin plus Vitamin D on ΔC-peptide Response at Month 24

ΔC-peptide response was defined as either having an increase in ΔC-peptide levels versus baseline or not decreasing more than 40%. The effect is given as the percentage­point difference between the groups at month 24. The 95% confidence intervals were estimated with the use of Wald approach. P value is obtained using a logistic-regression model adjusted for age at recruitment, sex and baseline levels.

Figure. S5.
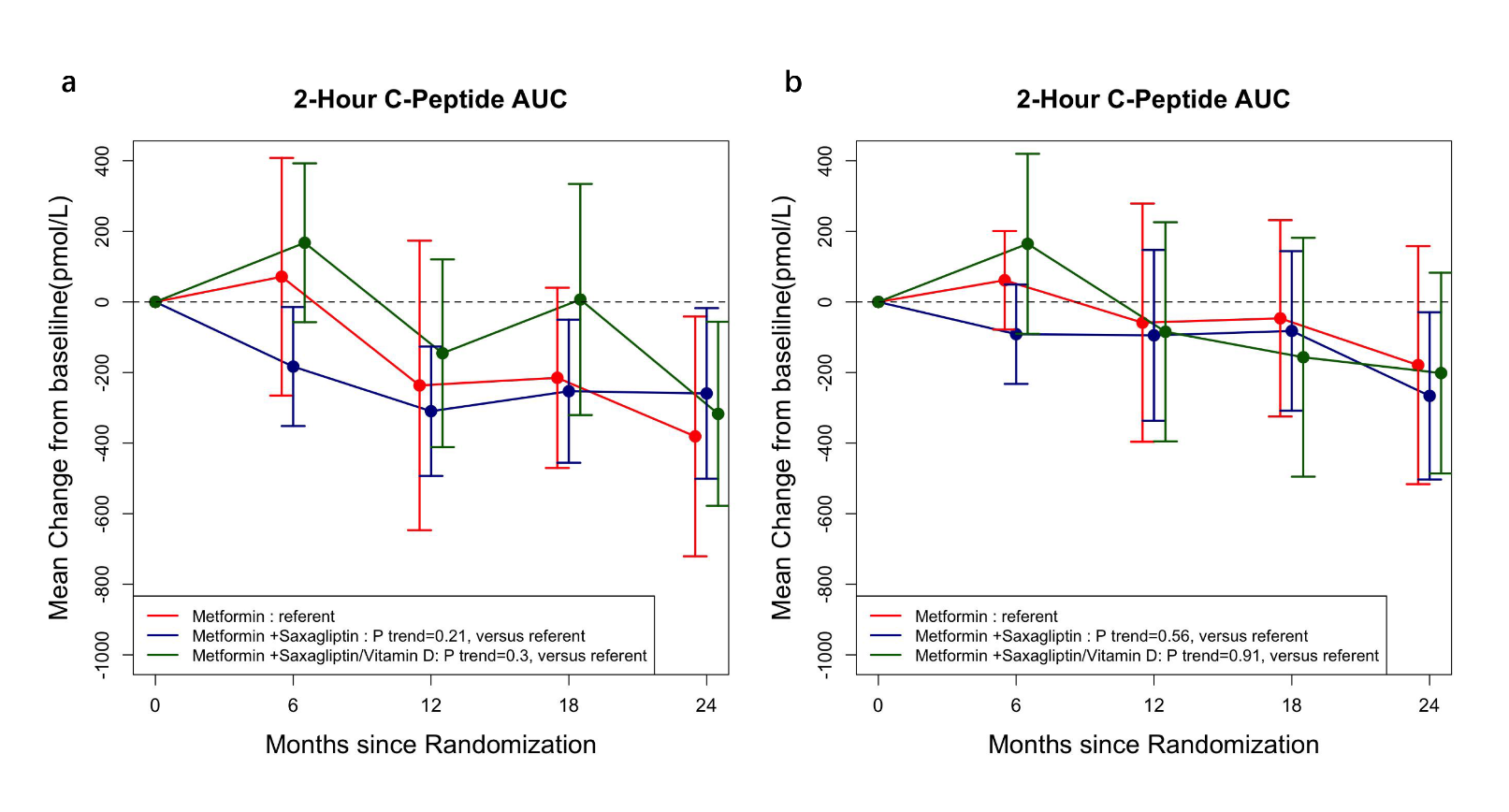
Effects of Saxagliptin and Saxagliptin plus Vitamin D on C-peptide AUC in patients with low and moderate GADA levels

(a) Mean change from baseline through 24 months in the mixed-meal-stimulated C-peptide AUC among participants with a low baseline GADA level of ≤180 U/mL in the Saxagliptin, Saxagliptin plus vitamin D and Conventional therapy groups.

(b) Mean change from baseline through 24 months in the mixed-meal-stimulated C-peptide AUC among participants with a moderate baseline GADA level of 180 U/mL to 577 U/mL in the Saxagliptin, Saxagliptin plus vitamin D and Conventional therapy groups.

Error bars indicate 95% confidence intervals; the mean values and their corresponding 95% confidence intervals are according to observed data. P for trend is obtained using mixed models for repeated measures. AUC=area under the curve; GADA=glutamic acid decarboxylase antibody.


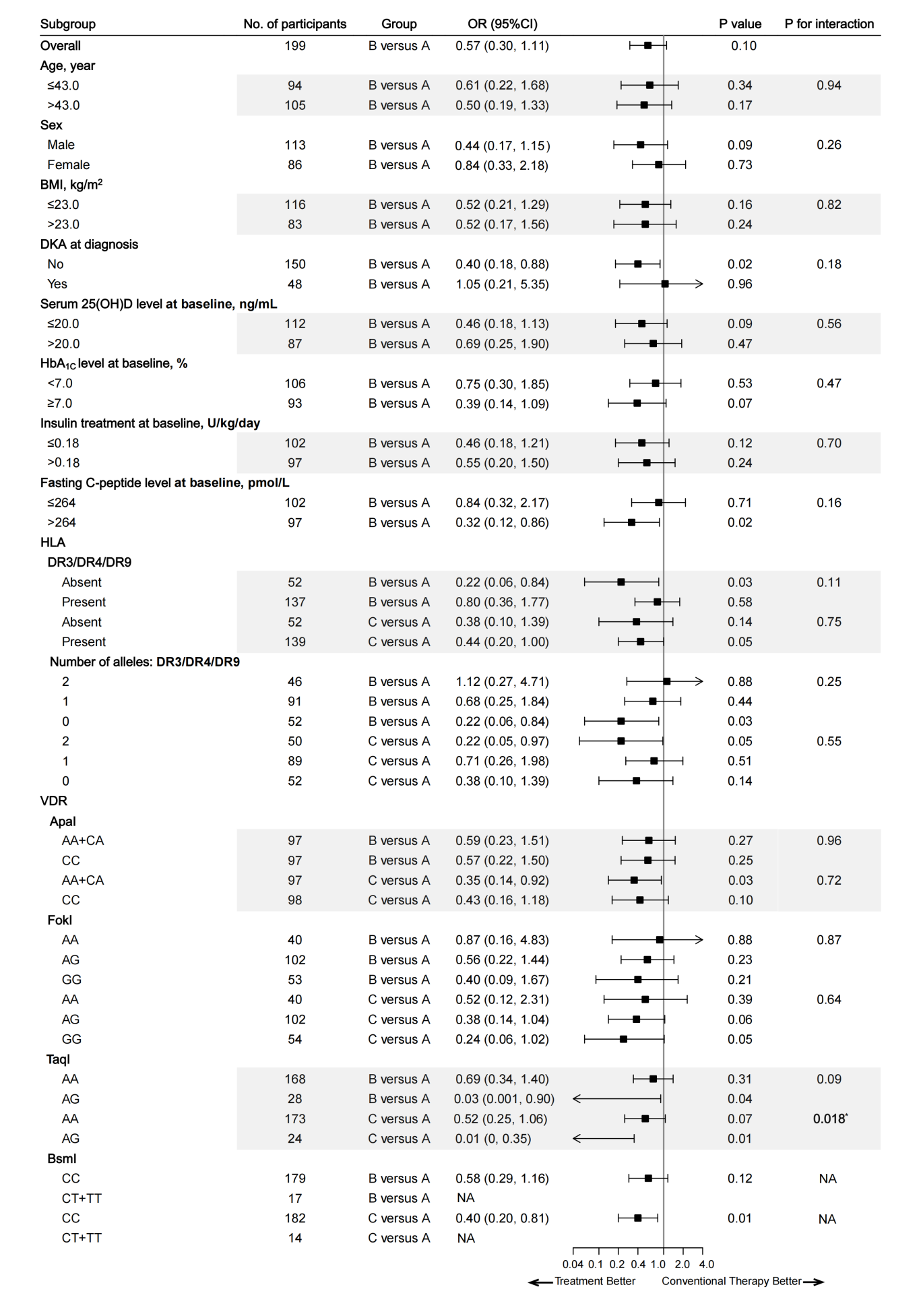
Figure. S6. Subgroup Analysis of Responses to Saxagliptin and Saxagliptin plus Vitamin D

The forest plot presents the odds ratios and their 95% confidence intervals for the changes from baseline through 24 months in the C-peptide AUC (two categories: low versus high, median cut-off point) in the Saxagliptin and Saxagliptin plus vitamin D group as compared with the conventional therapy, stratified by baseline factors (two categories of each). The cut-off points of age, insulin treatment and fasting C-peptide were median values. The logistic regression model was adjusted for age at recruitment, sex, baseline C-peptide (log[AUC+1]), baseline vitamin D concentration and DKA at diagnosis. Group A: Conventional therapy, Group B: Saxagliptin, Group C: Saxagliptin plus vitamin D; BMI=body mass index; DKA=diabetic ketoacidosis; AUC=area under the curve; HLA=human leukocyte antigen; VDR=vitamin D receptor. * P<0.05.

**Table S1. Effects of Saxagliptin and Saxagliptin plus Vitamin D on Endogenous β-cell Function**

|  | **2-hour C-peptide AUC (pmol/L): Mean (95% Confidence Interval)** | | | | |
| --- | --- | --- | --- | --- | --- |
|  | **Conventional therapy** | **Saxagliptin** | **P value** | **Saxagliptin plus Vitamin D** | **P value** |
| **Overall** |  |  |  |  |  |
| 0 month | 0 | 0 |  | 0 |  |
| 6 months | -85 (-233, 63) | -115 (-213, -17) | 0.83 | 76 (-41, 193) | 0.11 |
| 12 months | -263 (-444, -82) | -222 (-339, -105) | 0.33 | -135 (-269, -1) | 0.11 |
| 18 months | -280 (-437, -122) | -195 (-311, -79) | 0.47 | -107 (-255, 42) | 0.26 |
| 24 months | -419 (-601, -238) | -314 (-447, -181) | 0.28 | -276 (-399, -153) | 0.049 |
| **Mixed effect model*** |  |  | 0.14 |  | **0.01** |
|  |  |  |  |  |  |
| **GADA titer (U/mL):**  **Low level (≤ 180)** |  |  |  |  |  |
| 0 month | 0 | 0 |  | 0 |  |
| 6 months | 71 (-265, 408) | -183 (-352, -15) | 0.97 | 168 (-57, 393) | 0.58 |
| 12 months | -237 (-647, 174) | -310 (-493, -126) | 0.6 | -145 (-411, 121) | 0.60 |
| 18 months | -215 (-471, 40) | -253 (-456, -50) | 0.61 | 7 (-321, 334) | 0.44 |
| 24 months | -381 (-721, -41) | -259 (-501, -18) | 0.25 | -317 (-578, -56) | 0.26 |
| **Mixed effect model*** |  |  | 0.21 |  | 0.30 |
|  |  |  |  |  |  |
| **GADA titer (U/mL):**  **Moderate level (180 -≤378 [50^th^ percentile])** |  |  |  |  |  |
| 0 month | 0 | 0 |  | 0 |  |
| 6 months | 102 (-55, 258) | -72 (-245, 102) | 0.8 | 157 (-150, 464) | 0.77 |
| 12 months | -8 (-388, 373) | -6 (-307, 296) | 0.93 | -89 (-442, 263) | 0.95 |
| 18 months | -38 (-359, 284) | -32 (-297, 234) | 0.83 | -106 (-491, 279) | 0.64 |
| 24 months | -78 (-420, 263) | -273 (-544, -3) | 0.99 | -170 (-480, 141) | 0.90 |
| **Mixed effect model*** |  |  | 0.42 |  | 0.29 |
|  |  |  |  |  |  |
| **GADA titer (U/mL):**  **Moderate level (180 -≤577 [60^th^ percentile])** |  |  |  |  |  |
| 0 month | 0 | 0 |  | 0 |  |
| 6 months | 61 (-78, 201) | -91 (-232, 49) | 0.17 | 165 (-90, 420) | 0.99 |
| 12 months | -59 (-396, 279) | -95 (-337, 147) | 0.62 | -85 (-395, 226) | 0.86 |
| 18 months | -46 (-325, 232) | -82 (-308, 144) | 0.6 | -157 (-495, 182) | 0.39 |
| 24 months | -179 (-516, 158) | -266 (-503, -29) | 0.65 | -202 (-486, 83) | 0.78 |
| **Mixed effect model*** |  |  | 0.56 |  | 0.91 |
|  |  |  |  |  |  |
| **GADA titer (U/mL):**  **High level (> 378 [50^th^ percentile])** |  |  |  |  |  |
| 0 month | 0 | 0 |  | 0 |  |
| 6 months | -300 (-506, -95) | -76 (-234, 82) | 0.35 | -32 (-181, 118) | 0.04 |
| 12 months | -427 (-639, -215) | -226 (-408, -43) | 0.17 | -146 (-322, 30) | 0.03 |
| 18 months | -471 (-728, -214) | -201 (-375, -27) | 0.3 | -192 (-355, -29) | 0.10 |
| 24 months | -636 (-909, -364) | -371 (-572, -170) | 0.28 | -293 (-446, -139) | 0.03 |
| **Mixed effect model*** |  |  | 0.16 |  | **0.008** |
|  |  |  |  |  |  |
| **GADA titer (U/mL):**  **High level (> 577) 60^th^ percentile** |  |  |  |  |  |
| 0 month | 0 | 0 |  | 0 |  |
| 6 months | -328 (-559, -96) | -66 (-242, 110) | 0.26 | -75 (-223, 73) | 0.06 |
| 12 months | -439 (-663, -215) | -210 (-413, -7) | 0.18 | -161 (-327, 5) | 0.023 |
| 18 months | -520 (-799, -240) | -199 (-389, -10) | 0.023 | -174 (-304, -44) | 0.038 |
| 24 months | -607 (-895, -320) | -389 (-608, -169) | 0.024 | -293 (-434, -152) | 0.013 |
| **Mixed effect model*** |  |  | 0.10 |  | **0.001** |
|  |  |  |  |  |  |
| **GADA titer (U/mL):**  **High level (> 831 [70^th^ percentile])** |  |  |  |  |  |
| 0 month | 0 | 0 |  | 0 |  |
| 6 months | -361 (-651, -70) | -61 (-219, 97) | 0.28 | -49 (-230, 131) | 0.13 |
| 12 months | -471 (-760, -182) | -173 (-357, 12) | 0.34 | -191 (-422, 39) | 0.18 |
| 18 months | -513 (-888, -138) | -111 (-255, 33) | 0.35 | -204 (-378, -30) | 0.20 |
| 24 months | -618 (-983, -253) | -373 (-548, -199) | 0.24 | -328 (-510, -146) | 0.08 |
| **Mixed effect model*** |  |  | 0.10 |  | **0.02** |
|  |  |  |  |  |  |
| **GADA titer (U/mL):**  **High level (> 887 [75^th^ percentile])** |  |  |  |  |  |
| 0 month | 0 | 0 |  | 0 |  |
| 6 months | -380 (-724, -37) | -67 (-238, 105) | 0.26 | -27 (-243, 188) | 0.12 |
| 12 months | -532 (-869, -195) | -167 (-368, 33) | 0.16 | -204 (-483, 76) | 0.10 |
| 18 months | -572 (-1022, -121) | -137 (-274, 0) | 0.25 | -226 (-426, -26) | 0.18 |
| 24 months | -668 (-1082, -253) | -345 (-516, -173) | 0.11 | -384 (-591, -177) | 0.06 |
| **Mixed effect model*** |  |  | **0.04** |  | **0.02** |
|  |  |  |  |  |  |
| **GADA titer (U/mL):**  **High level (>955 [80^th^ percentile])** |  |  |  |  |  |
| 0 month | 0 | 0 |  | 0 |  |
| 6 months | -341 (-724, 42) | -38 (-249, 173) | 0.074 | 16 (-232, 265) | 0.014 |
| 12 months | -560 (-982, -139) | -141 (-394, 112) | 0.056 | -294 (-541, -47) | 0.056 |
| 18 months | -611 (-1150, -72) | -98 (-254, 58) | 0.041 | -194 (-457, 69) | 0.027 |
| 24 months | -638 (-1139, -137) | -329 (-549, -109) | 0.049 | -357 (-618, -97) | 0.022 |
| **Mixed effect model*** |  |  | **0.007** |  | **0.002** |

Mean change of AUC of C-peptide from baseline to 24 months was estimated using of a mix effects model of repeated measures of ANOVA, adjusted for age at recruitment, sex, time, treatment-by-time interaction, baseline C-peptide (log[AUC+1]), baseline C-peptide-by-time interaction, baseline serum 25(OH)D concentration and diabetic ketoacidosis (DKA) at diagnosis. AUC=area under the curve; GADA=glutamic acid decarboxylase antibody.

Table S2. Effects of Saxagliptin and Saxagliptin plus Vitamin D on Glycemic Control

|  | **HbA_1C_ (%): Mean (95% Confidence Interval)** | | | | |
| --- | --- | --- | --- | --- | --- |
|  | **Conventional therapy** | **Saxagliptin** | **P value** | **Saxagliptin plus Vitamin D** | **P value** |
| 0 month | 7.4 (7.0, 7.7) | 7.4 (7.0, 7.8) |  | 7.8 (7.3, 8.2) |  |
| 6 months | 7.1 (6.8, 7.4) | 6.8 (6.5, 7.1) |  | 6.9 (6.6, 7.2) |  |
| 12 months | 7.5 (7.1, 7.9) | 6.9 (6.6, 7.2) |  | 7.2 (6.8, 7.7) |  |
| 18 months | 7.7 (7.2, 8.1) | 7.4 (7.0, 7.8) |  | 7.3 (6.8, 7.7) |  |
| 24 months | 7.4 (7.0, 7.8) | 7.6 (7.2, 8.1) |  | 7.4 (7.0, 7.9) |  |
| Mixed effect model^a^ |  |  | 0.82 |  | 0.91 |

a.Mean values for HbA1c (glycated hemoglobin level) from baseline to 24 months, and P values were estimated by using of a mix effects model of repeated measures of ANOVA, adjusted for age at recruitment, sex, time, treatment-by-time interaction, baseline C-peptide (log[AUC+1]), baseline C-peptide-by-time interaction, baseline vitamin D concentration and diabetic ketoacidosis (DKA) at diagnosis.

Table S3. Effects of Saxagliptin and Saxagliptin plus Vitamin D on Insulin Requirement

|  | **Insulin dose (U/kg/day): Mean (95% Confidence Interval)** | | | | |
| --- | --- | --- | --- | --- | --- |
|  | **Conventional therapy** | **Saxagliptin** | **P value** | **Saxagliptin plus Vitamin D** | **P value** |
| **Mean change from baseline** |  |  |  |  |  |
| Baseline | 0 | 0 |  | 0 |  |
| 1 month | 0.0059 (-0.018, 0.03) | -0.024 (-0.051, 0.0027) |  | -0.0038 (-0.026, 0.019) |  |
| 3 months | -0.0061 (-0.036, 0.024) | -0.037 (-0.066, -0.0074) |  | -0.0016 (-0.029, 0.026) |  |
| 6 months | 0.0059 (-0.025, 0.037) | -0.035 (-0.066, -0.0042) |  | -0.0076 (-0.039, 0.024) |  |
| 12 months | 0.032 (-0.0017, 0.065) | -0.03 (-0.061, 0.018) |  | -0.0065 (-0.04, 0.027) |  |
| 18 months | 0.049 (0.0091, 0.089) | -0.017 (-0.049, 0.014) |  | 0.0056 (-0.03, 0.041) |  |
| 24 months | 0.072 (0.028, 0.12) | -0.01 (-0.047, 0.027) |  | 0.02 (-0.017, 0.057) |  |
| Mixed effect model^a^ |  |  | 0.0002 |  | 0.02 |
|  |  |  |  |  |  |
| **Absolute value** | **Insulin dose (U/kg/day): Mean value (SD)** | | | | |
| Baseline | 0.23 (±0.24) | 0.24 (±0.23) |  | 0.25 (±0.26) |  |
| 1 month | 0.24 (±0.23) | 0.22 (±0.21) |  | 0.24 (±0.26) |  |
| 3 months | 0.23 (±0.23) | 0.20 (±0.21) |  | 0.24 (±0.25) |  |
| 6 months | 0.24 (±0.24) | 0.20 (±0.21) |  | 0.24 (±0.27) |  |
| 12 months | 0.26 (±0.25) | 0.21 (±0.22) |  | 0.24 (±0.28) |  |
| 18 months | 0.28 (±0.27) | 0.22 (±0.22) |  | 0.25 (±0.28) |  |
| 24 months | 0.30 (±0.28) | 0.23 (±0.23) |  | 0.26 (±0.28) |  |

a. Mean change from baseline through 24 months and absolute value in daily insulin use, and P values were estimated by using of a mix effects model of repeated measures of ANOVA, adjusted for age at recruitment, sex, time, treatment-by-time interaction, baseline C-peptide (log[AUC+1]), baseline C-peptide-by-time interaction, baseline vitamin D concentration and diabetic ketoacidosis (DKA) at diagnosis.
