# Supplementary material for: Combination therapy with saxagliptin and vitamin D for the preservation of β-cell function in adult-onset type 1 diabetes: a multi-center, randomized, controlled trial
Source: Signal Transduct Target Ther. 2023 Apr 20;8:158. doi: 10.1038/s41392-023-01369-9 (PMC10115841; doi:10.1038/s41392-023-01369-9)
Supplement: Supplementary file 2 — Study Protocol [file 41392_2023_1369_MOESM2_ESM.pdf]

Clinical Study Protocol

Drug Substance	saxagliptin and vitamin D3
Study Code	2015BAI12B13
Edition Number	4.0-2
Date	14 Dec. 2017

A randomized controlled, open-label, multi-center study with 104-week fixed dose of saxagliptin or (and) vitamin D3 assessing protective effects on beta cell function in latent autoimmune diabetes in adults (LADA) treated with Metformin (and insulin)

Sponsor: ***The Second Xiangya Hospital of*** **Central South University**

PROTOCOL SYNOPSIS

A randomized controlled, open-label, multi-center study with 104-week saxagliptin or(and) vitamin D3 assessing protective effects on beta cell function in latent autoimmune diabetes in adults (LADA) treated with Metformin (and insulin)

Principal Investigator

Zhiguang Zhou, Xiang Yan, The Second Xiangya Hospital, Central South University

Co-Investigator (Co-PI):

Xia Li, Yufei Xiang, Chao Deng, The Second Xiangya Hospital, Central South University

Sub-Investigator (Sub-I):

Study site(s): 20-45 (China)

Number of subjects planned: 300

**Study period**	
Estimated date of first subject enrolled	Jan 2016
Estimated date of last subject enrolled	Apr 2017
Estimated date of last subject completed	May 2019
Estimated date of finalized study report	Dec 2019

Study design

This is a multi-center, open-label, 1:1:1 randomized controlled trial, compromised of a 6-week run-in period and the 104-week treatment period.

Target population：LADA patients signed an informed consent (the definition of LADA subjects please refer to the inclusion standards of the study) ; aged 18-70 years old; duration should be less than 4 years.

Randomize all subjects who meet all eligibility criteria and do not meet any of the exclusion criteria into three groups by a central dynamic random method: metformin (and insulin) , metformin (and insulin) + saxagliptin , metformin (and insulin) + saxagliptin + vitamin D3.

The main purpose of this study: evaluate whether saxagliptin (and vitamin D3) as add-on therapy to metformin (and insulin) can better protect islet beta cell function than metformin (and insulin).

Safety objective: Adverse events, serious adverse events, blood glucose, blood routine, serum creatinine, serum calcium and 25 (OH) D monitoring.

Objectives

Primary Objective:	Primary Outcome:
1. To evaluate the efficacy of adjunctive saxagliptin to metformin (and insulin) on beta cell function in compared with metformin (and insulin) in LADA patients.<br>1. To evaluate the efficacy of adjunctive saxagliptin combined with vitamin D3 to metformin (and insulin) on β-cell function compared with metformin (and insulin) in LADA patients.	Absolute changes from baseline in fasting C-peptide levels at week 104.
**Secondary Objective**	**Secondary Outcome:**
1. To evaluate the efficacy of adjunctive saxagliptin (and vitamin D3) to metformin (and insulin) on β-cell function in compared with metformin (and insulin) in LADA patients.<br>2. To evaluate the efficacy of adjunctive saxagliptin (and vitamin D3) to metformin (and insulin) on glycemic control /insulin-sparing/changes of autoantibody titer in compared with metformin (and insulin) in LADA patients.	1. Absolute changes from baseline in fasting C-peptide levels at week 26, 52 and 78.<br>2. Absolute changes from baseline in C-peptide at 60-min and 120-min (AUC) during a mixed-meal tolerance test at week 26, 52, 78 and 104.<br>3. The proportion of subjects with increased (decreased, unchanged) fasting or post-stimulus C-peptide level compared with baseline after 104 weeks of treatment.<br>4. The proportion of subjects with increased C-peptide pre-and post mixed-meal tolerance test (Delta C-peptide) after 104 weeks of treatment.<br>5. Changes of HbA1c levels from baseline

	at week 26, 52, 78 and 104.<br>6. The proportion of participants responding to hypoglycemic agents (defined as HbA1c<7%) after 104 weeks of treatment.<br>7. Changes of average daily insulin dose from baseline at week 26, 52, 78 and 104.<br>8. Changes of GADA titers from baseline at week 52 and 104.<br>9. Absolute changes of body weight and BMI Level from baseline at week 26, 52, 78 and 104.
**Safety Objective:**	**Outcome Measure:**
To assess the safety of saxagliptin (and vitamin D3) in LADA patients treated with metformin (and insulin).	AEs/SAEs, especially severe hypoglycaemia and potential hypercalcemia;<br>Vital signs;<br>Collection of clinical/chemistry parameters;<br>ECGs.
**Exploratory Objective:**	**Exploratory outcomes:**
1. To evaluate the add-on effect of vitamin D3 on saxagliptin compared with saxagliptin monotherapy in LADA patients on the following factors:<br>a. Protection of β-cell function<br>b. Effect on glycaemic control<br>c. Effect on insulin-sparing<br>d. Effect on autoantibody titers<br>2. To investigate the relationship between genes and effects of saxagliptin and vitamin	1. Absolute changes from baseline in C-peptide at fasting, 60-min and 120-min (AUC) during a mixed-meal tolerance test at week 26, 52, 78 and 104.<br>2. The proportion of subjects with increased (decreased, unchanged) fasting or post-stimulus C-peptide level compared with baseline after 104 weeks of treatment.

D3 in LADA patients treated with metformin (and insulin).	3. The proportion of subjects with increased C-peptide pre-and post mixed-meal tolerance test (Delta C-peptide) after 104 weeks of treatment.<br>4. Changes of HbA1c levels from baseline at week 26, 52, 78 and 104.<br>5. The proportion of subjects responding to hypoglycemic agents (defined as HbA1c<7%) after 104 weeks of treatment.<br>6. Changes of average daily insulin dose from baseline at week 26, 52, 78 and 104.<br>7. Changes of GADA titers from baseline at week 52 and 104.<br>8. HLA-DQ, DR and vitamin D receptor (VDR) gene polymorphism and immune repertoire polymorphism of patient before and after the treatment.

Investigational product, dosage and administration

Basic Medication:

First-line medication: Metformin, orally taken

- 1.5g per day (adjust the dose between 1-1.7g per day according to subject's specific situation).
- Investigators will provide guidance towards the brand, form, dosage and frequency.

Second-line medication: Insulin, subcutaneous injection

- Daily dose individualized.
- Investigators will provide guidance towards the brand, type, dosage and frequency.
- Insulin initiation suggestions: from the screening periods, if the subject's HbA1c≥7%, the researchers can decide whether to initiate insulin therapy (metformin is used in the meantime) for the prompt control of blood glucose (HbA1c≤7%).
- Insulin adjustment recommendations:

➢ Investigators can adjust the insulin dosages, change the insulin forms and stop the injections based on their own clinical experience, according to the subject 's blood glucose, physical conditions and other indicators.

➢ First reach the general blood glucose control level, then the ideal level, until the target level or the best appropriate level according to investigators.

➢ To avoid hypoglycemia first, then control hyperglycemia.

Investigational Medication:

Saxagliptin: 5mg per pill, orally taken, 1 pill per time, 1 time per day, take after randomization.

Vitamin D3: 400IU per capsule, orally taken, 5 capsules per time, once a day, take after randomization.

Statistical methods

All data are entered into SAS 9.3 software (SAS Institute Inc., Cary, NC, USA). The measurements will be calculated mean, standard deviation, median, quartile, minimum, and maximum. Categorical variables will describe as frequency and percentage. Changes from baseline in primary and secondary endpoints will be analyzed using a covariance pattern model, with treatment as classification variables and baseline value as a covariate. Point estimates and 95% confidence intervals (CIs) for the mean change within each treatment group, as well as for the differences in mean change between the two groups will be estimated. Differences in categorical variables will be analyzed using Pearson X^2, CMH X^2 or Fisher probabilities. For primary outcomes, $p< 0.025$ will be considered as significant. Other outcomes $p<0.05$ will be considered as significantly.

Adverse events (AEs) will be summarized. The incidence of adverse events will be summarized by body system organ class and preferred term. Analyses for safety and tolerability endpoints will be summarized using descriptive statistics for continuous variables or frequency counts and percentages for categorical variables. All conclusions will be based on final analysis rather than the interim analysis.

Sample Size

The sample size calculations were performed based on the primary variable "absolute change in fasting C-peptide from baseline to week 104".

Because there is no study for the effect of saxagliptin combined with vitamin D3 on β-cell function of diabetes patients, the analysis for power of test in this study is based on the articles about sitagliptin combined with insulin compared to insulin alone on the β-cell function of LADA patients within one year. Since our hypothesis are that saxaliptin will increase the protective effect on β-cell function in LADA treated with metformin (and insulin), and vitamin D3 added on saxaliptin will increase the protective effect on β-cell function in LADA treated with metformin (and insulin), the estimation for sample size is according to the comparison of two sample means. The means of effect of conventional treatment and sitagliptin on fasting C-peptide (the change of fasting C-peptide from baseline at 1 year) which is the indication of β-cell function are 149.2, 108.4, respectively, the standard deviations are 84.5, 52.8 respectively. Set $\alpha=0.025$ as reaching the statistical difference, and set $\beta=0.1$, the power of test is 90%. The minimum sample size is 76. According to calculation with the drop-out rate, which is approximate 20%, 95 patients per group are needed. We set 100 cases per group.

TABLE OF CONTENTS

PROTOCOL SYNOPSIS....2
1. INTRODUCTION.... 16
1.1 Background.... 16
1.2 Research hypothesis.... 18
1.3 Rationale for study design, doses and control groups.... 18
1.3 Benefit/risk and ethical assessment.... 20
2. STUDY OBJECTIVES AND THERAPEUTIC EVALUATION.... 24
2.1 Primary objectives and therapeutic indicators.... 24
2.2 Secondary objectives and therapeutic indicators.... 24
2.3 Safety objectives and evaluating indicators.... 25
2.4 Exploratory objectives and evaluating indicators.... 26
3. STUDY DESIGN.... 27
4. STUDY SUBJECT.... 28
4.1 Inclusion criteria/Exclusion criteria:.... 28
4.2 Subject enrollment.... 29
4.3 Method of Randomization.... 29
4.4 Procedures for incorrectly enrolled or randomized subjects.... 29
5. INVESTIGATIONAL DRUGS.... 30
5.1 Features of investigational product(s).... 30
5.2 Treatment regimens.... 30
5.3 Packaging, labeling and storage of the investigational drugs.... 31
5.4 Compliance Evaluation.... 32
5.5 Prohibition of drugs.... 33
5.6 Permitted combination medication.... 33
6. STUDY PLAN.... 34
6.1 Visit schedule.... 34
6.2 Measurements during the study.... 34
6.3 Time of procedure.... 35

6.4 Visiting time....38
6.5 Screening Failure....47
6.6 Discontinuation of Study....47
7. STUDY ASSESSMENTS....48
7.1 Efficacy assessments....48
7.2 Safety assessments....48
7.3 Other assessments....52
7.4 Collection and processing of biological samples....53
8. Safety reporting and medical management....53
8.1 Definition of adverse events (AE) and treatment plans....54
8.2 Definition of serious adverse events (SAEs)....55
8.3 Causality assessment of (severe) adverse events....57
8.4 Treatment on severe nausea and vomiting....58
8.5 Treatment on hypoglycemia....58
8.6 Treat on hypercalcemia....59
8.7 Treatment on vitamin D intoxication....60
8.8 Overdose....61
8.9 Pregnancy....61
9. STATISTICAL ANALYSIS....62
9.1 Statistical considerations....62
9.2 Sample size estimation....62
9.3 Definitions of analysis sets....63
9.4 Outcome measures for analysis....64
10. STUDY AND DATA MANAGEMENT....66
10.1 Monitoring of the study....66
10.2 Study timetable and end of study....68
10.3 Data management....69
11. ETHICAL AND REGULATORY REQUIREMENTS....69
11.1 Ethical conduct of the study....69
11.2 Subject data protection....70
11.3 Ethics and regulatory review....70
11.4 Informed consent....70
11.5 Changes to the protocol and informed consent form....71
12. QUALITY CONTROL AND QUALITY ASSURANCE....72

12.1 Monitoring.......... 72
12.2 Audits.......... 73
12.3 Inspections.......... 73
13. LIST OF REFERENCES.......... 74

LIST OF TABLES

Table 1 Visit Design and Visit Windows 26

LIST OF FIGURES

Figure 1 Study flow chart.......... 36

LIST OF APPENDICES

Appendix 1: Signatures
Appendix 2: List of research center
Appendix 3: Samples collection, transport and test results report procedures

List of abbreviations and definition of terms

The following abbreviations and special terms are used in this study Clinical Study Protocol.

Abbreviation or special term	Explanation
AACE	American Association of Clinical Endocrinologists
AEs	Adverse events
ALB	Serum albumin
ALT	Alanine transaminase
AST	Aspartate transaminase
ANCOVA	Analysis of covariance
AUC	Area under the curve
BMI	Body Mass Index
BUN	Blood Urea Nitrogen
CDS	Chinese Diabetes Society
CFDA	China Food and Drug Administration
CI	Confidence intervals
Cr	Creatinine
CRF	Case report form
CRO	Contract research organization
CSA	Clinical Study Agreement
DBIL	Direct Bilirubin
DBP	Diastolic blood pressure
DMC	Data monitoring committee
DPP-IV	Dipeptidyl peptidase IV
EC	Ethics Committee
ECG	Electrocardiogram
eGFR	Estimated glomerular filtration rate
FAS	Full analysis set
FCP	Fasting serum C-peptide
FPG	Fasting Plasma Glucose
GADA	Glutamic acid decarboxylase antibodies
GLP-1	Glucagon-like peptide
GMP	Good Manufacturing Practice

**Abbreviation or special term**	**Explanation**
HbA1c	Haemoglobin A1c
HDL-C	High-density lipoprotein cholesterol
HLA	Human Leucocyte Antigen
IASP	Islet Autoantibody Standardization Program
ICH	International Conference on Harmonization
IDS	Immunology of Diabetes Society
IP	Investigational Product
ITT	Intention-to-treat
LADA	Latent autoimmune diabetes in adults
LDL-C	Low-density lipoprotein cholesterol
LOCF	Last observation carried forward
MMRM	Mixed model repeated measures
MMTT	Mixed-meal tolerance test
NOD	Non-obese diabetic
PCR	Polymerase chain reaction
PP	Per Protocol
PPG	Postprandial pulse glucose
RCT	Randomized Controlled Trial
SAEs	Serious adverse events
SAP	Statistical Analysis Plan
SBP	Systolic blood pressure
SMBG	Self-monitoring of blood glucose
SOP	Standard operation
SS	Safety Analysis Set
SU	Sulfonylureas
T1D	Type 1 diabetes
T2D	Type 2 diabetes
TBIL	Total bilirubin
TC	Total Cholesterol
TG	Triglyceride
TZD	Thiazolidinedione
UA	Uric Acid
UACR	Urine albumin-to-creatinine ratio
VDR	Vitamin D receptor

**Abbreviation or special term**	**Explanation**
25(OH)D	25-hydroxy vitamin D

1. INTRODUCTION

1.1 Background

At present, China has become the country with the largest number of diabetic patients, and the disease burden of diabetes keeps increasing, especially in developing countries. Using the China Disease Surveillance System death cause monitoring and China's chronic disease and its risk factors monitoring data, it is estimated that the 2010 China Diabetes Disability Life Year (DALY), the Chinese population of diabetes per thousand people DALY value of 19.12 person-years, mainly in the residents aged 15 to 79 years, of which the highest population was 45 to 60 years old, for whom the labor force burden is higher [(1)]. This result stems from the fact that diabetes increases the risk of heart disease and stroke over time. 50% of people with diabetes die from cardiovascular and cerebrovascular diseases (mainly heart disease and stroke). Foot neuropathy (nerve damage) combined with reduced blood flow increases the risk of foot ulcers, infections, and eventually amputation. Diabetic retinopathy is one of the main causes of blindness, which is the result of long-term accumulation of small blood vessels in the retina. 1% of global blindness can be attributed to diabetes. One of the main causes of kidney failure is diabetes as well. The prevalence of osteoporosis in patients with type 1 diabetes is higher than in the general population. The total risk of death is at least doubled for diabetic patients than their peers without diabetes. The risk of macrovascular, microvascular complications and death in patients with T1D was also significantly higher than in non-diabetic patients. Therefore, measures to prevent and treat diabetes are urgently needed. Adult-onset latent autoimmune diabetes (LADA) is considered to be a special phenotype of diabetes. In fact, LADA belongs to a form of T1D, which is caused by autoimmune destruction of islet β-cells induced by environmental factors based on genetic susceptibility background. LADA has some clinical features of T2D at onset, which is characterized by slow and latent onset, and is easily misdiagnosed as T2D due to the slow deterioration of β-cell function [(2,3)]. A variety of antibodies can be detected in the serum of patients, such as glutamic acid decarboxylase antibody (GAD-Ab), protein tyrosine phosphatase ELISA (IA-2A), zinc The transporter 8 antibody (ZnT8-Ab) and the like, among which the sensitivity of GAD-Ab is the highest [(4)]. T1D often combines with other autoimmune diseases, such as Hashimoto thyroiditis, Graves disease, celiac disease,

Addison disease, vitiligo, etc. For patients with atypical clinical manifestations, anti-thyroglobulin antibody and anti-thyroid peroxidase can be detected. Antibodies, anti-tissue glutamine transferase antibodies, and the like are screened. According to a national, multi-center, hospital-based cross-sectional study led by the Second Xiangya Hospital of Central South University, there are about 6 million autoimmune diabetes patients in China, that is, China is also the country with the largest population with autoimmune diabetes in the world(5).

Since the residual β-cell function in LADA declined much more slowly than that in classic T1D, LADA may serve as an appropriate human model for investigation of the effect of potential agents for β-cell preservation. The goals of treatment for LADA are suppression of autoimmune β cell destruction, preservation of islet function and prevention of diabetic complications. When we choose the medications for LADA, the efficacy, safety, cost and availability should be taken into considerations. The ideal agents for clinical setting may have both of the ability to promote β cell proliferation and modulate immune system.

Among oral hypoglycemic agents, dipeptidyl peptidase IV (DPP-IV) inhibitors are promising in improving β cell function in T2D patients. Clinical studies have demonstrated that either DPP-IV inhibitors alone or in combination with other drugs can reduce the patient's HbA1c and improve the function of pancreatic β-cells. DPP-IV, as a widely expressed enzyme, plays an important role in the development, activation and immune regulation of T cells. Many studies in animal models demonstrated that DPP-IV inhibitors are effective in some organ-specific autoimmune disease. Our previous randomized-controlled pilot study showed that DPP-4 inhibitors could significantly improve islet β-cell function in patients with LADA (6).

Vitamin D, a potent regulator of calcium and phosphate metabolism, has recently been shown to possess immunomodulatory properties. Vitamin D receptor (VDR) is distributed widely in both islet cells and lymphocytes, suggesting that vitamin D has a regulatory role on the immune system and islet cells. Evidence (including those from NOD mice, islet transplantation and patients with type 1 diabetes) in recent years have demonstrated that vitamin D could have a preventive effect on autoimmune diabetes. We have carried out a randomized-controlled pilot study (7), which suggested that 1, α-vitamin D3 and insulin combination could preserve pancreatic β-cell function in patients with LADA. Besides,

vitamin D3 is affordable, easy to access, and has few side effects. If we could prove the efficacy of vitamin D3 in β-cell function or glucose control on LADA population, more patients with LADA can benefits from the standardized treatment of vitamin D3.

1.2 Research hypothesis

- 1） Saxagliptin will have protective effect on β cell function in LADA treated with metformin (and insulin), absolute changes from baseline in fasting C-peptide levels at week 26, 52, 78 and 104 will be less than those treated with metformin (and insulin) alone.
- 2） Saxagliptin and vitamin D3 will have protective effect on β cell function in LADA treated with metformin (and insulin), absolute changes from baseline in fasting C-peptide levels at week 26, 52, 78 and 104 will be less than those treated with metformin (and insulin) alone.

1.3 Rationale for study design, doses and control groups

The aim of LADA management are to decrese autoimmune β-cell destruction, to protect β-cell function and to prevent diabetes complications. The status quo is the lack of multi-center evidence-based effective intervention for LADA, taking the high prevalence of LADA in China into consideration, it’s necessary to conduct a multi-center Randomized Controlled Trial (RCT) to explore the effective interventions for patients with LADA. The efficacy, safty, cost and availability of agents should be considered comprehensively.

Metformin is the most widely used oral anti-diabetic drug worldwide, which has advantages in cost-effectiveness. It acts directly on the glucose metabolism, promotes anaerobic glycolysis, increases the glucose uptake and usage in muscle, fat and other peripheral tissues, and thus protects the impaired islet β-cell function from further damage. Metformin is beneficial in long-term control of diabetes—the inhibition of intestinal absorption of glucose and hepatic gluconeogenesis; the reduction of hepatic glucose output; the reduction in blood glucose and glycated hemoglobin in diabetic patients. For type 1 or type 2 diabetes, metformin combined with insulin may increase the

glucose-lowering effect of insulin, reduce insulin dosage and thus prevent hypoglycemia. Recent studies have also found that metformin has anti-aging, anti-tumor, cardiovascular events risk lowering and life prolonging effects (8-10). Many guidelines and consensus abroad regard metformin as the first choice for T2D drug treatment (11-12). As long as the patient can tolerate it and there is no drug contraindication, metformin should be continuously used in combined therapy strategy. LADA has similar early clinical manifestations with T2D, especially in insulin resistance (4). Although LADA is not an indication for metformin yet, no research has suggested that metformin cannot be used in LADA patients. Therefore, this study will try using metformin as a first-line basic medication, under the informed consent of patients.

Insulin has been widely used in clinic for nearly 100 years. Its remarkable glucose lowering effect has been approved in numerous patients. Evidence-based strategies for preservation of β cell function in LADA are rare, but insulin therapy had been shown to have a beneficial effect in pilot studies (13,14). Thus, this study will use insulin as a second-line basic medication. Insulin will be added to treatment when target glucose level cannot be reached, or clinicians find it difficult to control the glucose by metformin alone. To meet the individualized needs for daily life and work according to patients conditions, researchers could choose the most suitable insulin type, dosage and injection frequency for each subject, in order to lower the incidence of hypoglycaemia and maintain an ideal glucose level safely.

DPP-IV, as a widely expressed enzyme, plays an important role in the development, activation and immune regulation of T cells. A randomized double-blind placebo-controlled parallel trial in the UK showed that when saxagliptin 5 mg/day or placebo were added to insulin or insulin plus metformin treated patients with T2DM for 52 weeks, the saxagliptin group had more stable and long-lasting glucose-lowering effect, and the patients were better tolerated (15) .Our previous randomized-controlled pilot study showed that DPP-4 inhibitors could significantly improve islet β-cell function in patients with LADA (6). Clinical studies have demonstrated that either saxagliptin (5mg/d) alone or in combination with other drugs can reduce the patient's HbA1c and improve the function of pancreatic β cells. In this study we will use saxagliptin (5mg/d) plus metformin (and insulin) to treat LADA patients, and expect to see the better β cell protective function.

Vitamin D receptor (VDR) is distributed widely in islet cells and lymphocytes, suggesting that vitamin D has a regulatory role on the immune system and islet cells. Evidence (including those from NOD mice, islet transplantation and patients with type 1 diabetes) in recent years have demonstrated that vitamin D could have a preventive effect on autoimmune diabetes. A Northern Finland Birth Cohort study reported 86% of type 1 diabetes risk reduction was observed if the dose of Vitamin D given to the infant was at least 2000IU per day (8). A randomized double-blind placebo-controlled trial from Brazil with newly diagnosed T1D showed that the addition of vitamin D3 2000IU/day in the diabetes treatment for 18 months had immunomodulatory protective effects, and could also delay the decrease of remaining β-cell function (18) without safety issues. In 2011, the American Endocrine Society clinical practice guidelines recommend that vitamin D3 2000 IU/d is the maintenance dose for the American adults who are vitamin D deficient after loading dose treatment (19). A clinical trial including 340 cases of Lebanese adolescent subjects showed that vitamin D3 2000 IU/d treatment for a year could achieve the vitamin D requirements safely (20) . There are also some clinical trials of vitamin D3 in China chose 2000 IU/d as the dosage (21,22) while some others chose 4000 IU/d (23). The doses of saxagliptin and vitamin D3 are determined according to our previous studies and are supported by domestic and abroad studies. A previous randomized-controlled pilot study by our group suggested that 1,α-vitamin D3 and insulin combination could preserve pancreatic β-cell function in patients with LADA(7). Moreover, vitamin D3 is affordable, easy to access, and has few side effects. In this study, vitamin D3 2000IU/day will be used in combination of saxagliptin 5 mg/day as an add-on therapy to metformin (and insulin).

1.3 Benefit/risk and ethical assessment

When choosing therapy, not only need we consider the treatment effect, but also we should always pay attention to patient's safety. Not only should we carefully monitor the side effects of medications, but also try our best to decrease the potential risk.

The main side effects of metformin are gastrointestinal reactions, such as anorexia , nausea , abdominal pain , diarrhea, etc. The symptoms can be relieved if the medication is taken during or after a meal, and usually a withdrawal is not necessary. If metformin is prescribed improperly or large dose is taken, it may lead to lactic acidosis (although the incidence is very low). In addition, long-term usage of metformin may reduce the

absorption of vitamin B12. In general, metformin is safe, Metformin has no direct damaging effect on the kidney. But there will be drug accumulations when patients have already had kidney damage. In this study, renal function is monitored regularly so that metformin would be reduced or stopped when necessary [11] .

Insulin has been so widely used in diabetic and non-diabetic patients since 1920s. Its remarkable glucose lowering effect has been approved in numerous studies in LADA patients. Hypoglycaemia is the main concern when treated with insulin, but can be decreased or avoided by carefully titrate the dosage and frequently monitoring blood glucose. Other side effects are rare. We tried to explore the optimized strategy based on metformin and insulin for LADA.

Six inportant phase 3 trials of saxagliptin has shown statistically significant and clinically relevant glucose lowering effect with a safety and tolerability profile similar to placebo. There are two placebo-controlled studies as monotherapy, three placebo-controlled studies as add-on agent (add-on to thiazolidinedione [TZD], sulphonylurea (SU), and metformin, respectively), and one active controlled study where saxagliptin was given as initial treatment in combination with metformin. The key results of these studies are: treatment with saxagliptin resulted in consistent, clinically meaningful and statistically significant reductions in HbA1C, fasting plasmaglucose (FPG), and postprandial plasma glucose (PPG), as well as achievement of treatment targets for HbA1C. Beneficial effects were demonstrated across subgroups of demographic and baseline characteristics, saxagliptin treatment was associated with sustained glycemic control relative to the placebo; Saxagliptin was well tolerated in all clinical trials. The majority of reported adverse events (AEs) were mild and did not require treatment discontinuation. The safety profile was generally consistent when saxagliptin was given as monotherapy, as add-on combination treatment to metformin, SU, or TZD, or as initial therapy in combination with metformin.

Vitamin D possesses immunomodulatory properties. Vitamin D receptor (VDR) is distributed widely in islet cells and lymphocytes, suggesting that vitamin D has a regulatory role on the immune system and islet cells. Evidence in recent years has demonstrated it could have a preventive effect on autoimmune diabetes. We have carried out a randomized-controlled pilot study [7], which suggested that 1, α-vitamin D3 and insulin combination could preserve pancreatic β-cell function in patients with LADA.

The U.S. medicine institute of food and nutrition committee concluded some study evidence from vitamin D which covers different dose range (800 – 300000 IU/day) and duration (a few months to years). Vitamin D supplement < 10000IU/day scarely lead to poisoning. However, undiagnosed primary hyperparathyroidism patients can be found when given vitamin D treatment. Genetic abnormal metabolism or other comorbidities like chronic kidney disease, granuloma or hyperparathyroidism, can make the patients susceptible to vitamin D supplementation. There is no clear evidence showing the relation between the upper safety level of 25(OH)D and hypercalcemia. Most experts believed that when 25(OH)D<150 nmol/L, generally there is no need to worry about the side effects of vitamin D (19). The results from studies about vitamin D dose response differ from study to study. Among these studies, the UV exposure in summer is the important confounding factor.

The current evidence is not sufficient on the time to monitor the response to vitamin D treatment,(25). According to the pharmacokinetics of 25(OH)D, it should be monitored within 1 month of the final full load. Most studies have shown that 25(OH)D can reach a new steady state after about 3 months, but some studies have found that 25(OH)D has not reached steady state levels in 6 months after treatment. Therefore, some experts believe that one month after the vitamin D load supplementation, or after vitamin D supplementation, symptoms of subclinical primary hyperparathyroidism is revealed, and serum calcium should be corrected. There is no need to routinely measure 25(OH)D, which can result in wasted resources. However, considering that the dose of vitamin D used in this study is 2000 IU/day, exceeding the upper limit of 800 IU/day specified by the specification, researchers, ethics committee members and subjects will be very concerned about the safety of treatment;Different people have different sensitivities to vitamin D treatment, and some people may have increased sensitivity, which may increase the risk of poisoning or adverse reactions; and only one of the three groups received vitamin D. Is there a difference in the safety of the treatment among three groups? Is this difference related to different vitamin D levels? Regular measurements of 25(OH)D are needed to answer these questions.

The research team conducted a preliminary experiment in the early stage, including 10 normal volunteers with normal glucose tolerance, taking oral vitamin D3 2000 IU/day for 3 months. At the beginning of the study and at the 2nd, 4th, 8th and 12th week of the

study, 25(OH)D levels, serum calcium and phosphorus levels, and 24-hour urinary calcium and phosphorus excretion were monitored. The 25(OH)D level of all volunteers was within the safe range (<500 nmol/L), and there was no abnormality in serum calcium and phosphorus levels and 24-hour urinary calcium and phosphorus excretion.

Therefore, combined with domestic and foreign guidelines, existing research and our pre-experiments, it is concluded that vitamin D3 2000 UI/d is a safe dose and the possibility of poisoning is low.To ensure the safety of the study and detect vitamin D3 excessive promptly, we will measure vitamin D3 levels, serum calcium, phosphorus, 24-hour urinary calcium and phosphorus, and monitor urinary ultrasound before randomization and at 4, 12, 26, 52, 78 and 104 weeks after intervention. According to China's Vitamin D and Adult Skeletal Health Guidelines, which cited the United States guidelines, a potential vitamin D poisoning should be considered when 25(OH)D level persistently over 500 nmol/L(25). In this study, when 25(OH)D>250 nmol/L, the supplemental dose of vitamin D needs to be reduced to 800 IU/day. If patient develops hypercalcemia, physicians should look for possible causes, such as dehydration (most likely), mild primary hyperparathyroidism, high calcium intake, infection, etc., and provide treatment accordingly.

2. STUDY OBJECTIVES AND THERAPEUTIC EVALUATION

2.1 Primary objectives and therapeutic indicators

**Primary Objective:**	**Primary Therapeutic Indicator:**
1. To evaluate the efficacy of adjunctive saxagliptin to metformin (and insulin) on β -cell function in compared with metformin (and insulin) in LADA patients.<br>2. To evaluate the efficacy of adjunctive saxagliptin combined with vitamin D3 to metformin (and insulin) on β-cell function compared with metformin (and insulin) in LADA patients.	Absolute changes from baseline in fasting C-peptide levels at week 104.

2.2 Secondary objectives and therapeutic indicators

**Secondary Objective:**	**Secondary Therapeutic Indicator:**
1. To evaluate the efficacy of adjunctive saxagliptin (and vitamin D3) to metformin (and insulin) on β-cell function in compared with metformin (and insulin) in LADA patients .	1. Absolute changes from baseline in fasting C-peptide levels at week 26, 52 and 78<br>2. Absolute changes from baseline in C-peptide at 60-min and 120-min (AUC) during a mixed-meal tolerance test at week 26, 52, 78 and 104<br>3. The proportion of subjects with increased (decreased, unchanged) fasting or post-stimulus C-peptide level compared with baseline after 104 weeks of treatment
2. To evaluate the efficacy of adjunctive saxagliptin (and vitamin D3) to metformin	4. The proportion of subjects with increased C-peptide pre-and post

(and insulin) on glycemic control /insulin-sparing/changes of autoantibody titer in compared with metformin (and insulin) in LADA patients.	mixed-meal tolerance test (Delta C-peptide) after 104 weeks of treatment.<br>5. Changes of HbA1c levels from baseline and at week 26, 52, 78 and 104<br>6. The proportion of participants responding to hypoglycemic agents (defined as HbA1c<7%) after 104 weeks of treatment<br>7. Changes of average daily insulin dose from baseline and at week 26, 52, 78 and 104<br>8. Changes of GADA titers from baseline and at week 52 and 104<br>9. Absolute changes of body weight and BMI level from baseline and at week 26, 52, 78 and 104

2.3 Safety objectives and evaluating indicators

**Safety Objective:**	**Safety Indicator:**
To assess the safety of saxagliptin with or without vitamin D3 in LADA patients treated with metformin (and insulin)	AEs/SAEs, especially Severe hypoglycaemia and potential hypercalcemia<br>Vital signs<br>Collection of clinical chemistry/haematology parameters<br>ECGs.

2.4 Exploratory objectives and evaluating indicators

**Exploratory Objective**:	**Exploratory evaluating indicator:**
1. To evaluate the add-on effect of vitamin D3 on saxagliptin compared with saxagliptin monotherapy in LADA patients on the following factors:<br>a. Protection of β-cell function<br>b. Effect on glycaemic control<br>c. Effect on insulin-sparing<br>d. Effect on autoantibody titers<br>2. To investigate the relationship between genes and effects of saxagliptin and vitamin D3 in LADA patients treated with metformin (and insulin).	1. Absolute changes from baseline in C-peptide at fasting, 60-min and 120-min (AUC) during a mixed-meal tolerance test at week 26, 52, 78 and 104.<br>2. The proportion of subjects with increased (decreased, unchanged) fasting or post-stimulus C-peptide level compared with baseline after 104 weeks of treatment.<br>3. The proportion of subjects with increased C-peptide pre-and post mixed-meal tolerance test (Delta C-peptide) after 104 weeks of treatment.<br>4. Changes of HbA1c levels from baseline at week 26, 52, 78 and 104.<br>5. The proportion of subjects responding to hypoglycemic agents (defined as HbA1c<7%) after 104 weeks of treatment.<br>6. Changes of average daily insulin dose from baseline at week 26, 52, 78 and 104.<br>7. Changes of GADA titers from baseline at week 52 and 104.<br>8. HLA-DQ, DR and vitamin D receptor (VDR) gene polymorphism and immune repertoire polymorphism of patient before and after the treatment.

3. STUDY DESIGN

This is a multi-center, open-label, 1:1:1 randomized controlled trial, involving 20-45 centers among the country, and a total of 300 subjects, and is a prospective research comprising of a 104-week open-label intervention period.

On the basis of the inclusion/exclusion, eligible patients will be selected to be randomly allocated into groups and initiate the 104-week open-label intervention period. A central randomization system will be adopted, and the study population will be stratified based on the following: fasting C-peptide (FCP) ≧300 pmol/L and < 300 pmol/L; GADA titer ≧0.3 and <0.3 index; BMI ≧ 25 kg/m^2 and < 25 kg/m^2. Patients will be randomized into the following three groups: metformin (and insulin), metformin (and insulin)+saxagliptin, metformin (and insulin)+saxagliptin+vitamin D3 (Figure 1).

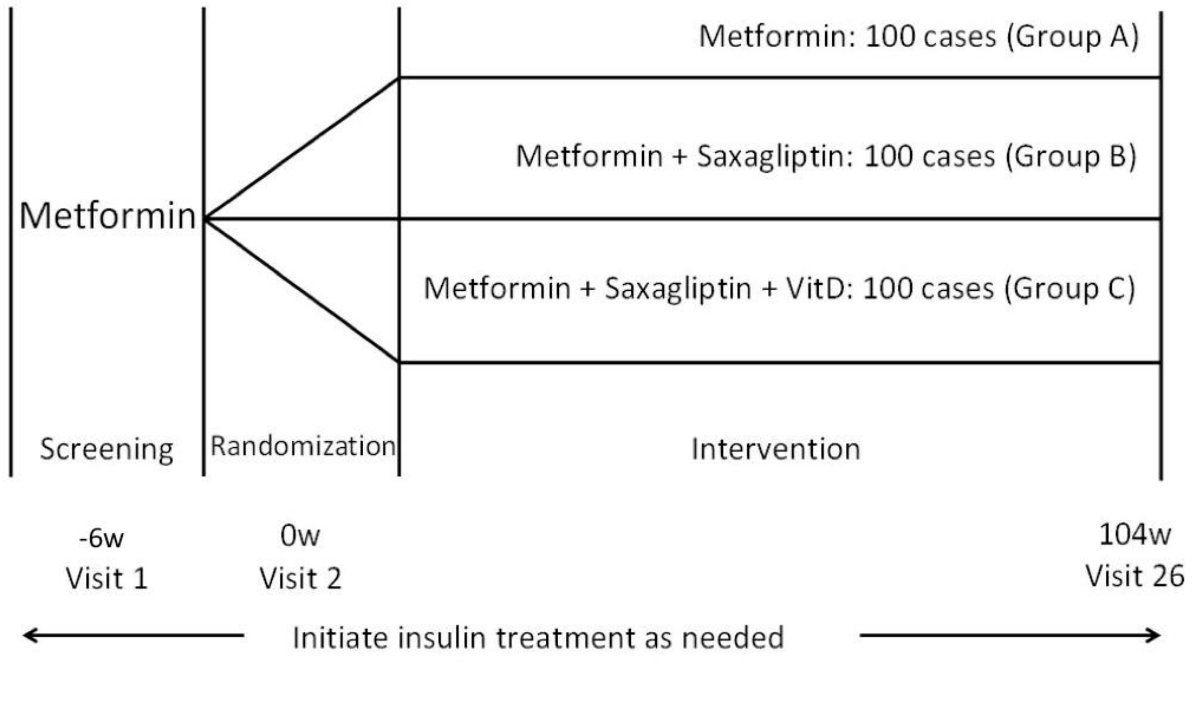

Figure 1. Study Flow Chart

4. STUDY SUBJECT

4.1 Inclusion criteria/Exclusion criteria:

Inclusion criteria:

1) Voluntarily joined with informed consent

2) The LADA patients to be included in this study are defined as:

   (1) Meet the 1999 WHO Diagnostic Criteria for Diabetes Mellitus;

   (2) Age at diagnosis ≧18 years old;

   (3) GADA positive;

   (4) Serum fasting C-peptide ≧100pmol/L or 2-hour postprandial C-peptide≧200pmol/L.

3) Age between18-70 years old

4) Duration of diabetes <4 years;

5) Outpatient or inpatient.

Exclusion criteria:

1) Women who are pregnant, intending to become pregnant during the study period, or currently lactating females;

2) Gestational diabetes mellitus or other specific types of diabetes;

3) Allergy to saxagliptin, vitamin D3 and excipient;

4) Treated with medications including DDP-IV inhibitors, GLP-1 analogues, GLP-1 receptor agonists, and TZDs in the past 8 weeks prior to randomization;

5) Patients with hypercalcemia (exceeding the upper limit of the reference range)

6) Treatment with systemic corticosteroids therapy (oral or intravenous) consecutively for over 7 days in the past 6 months;

7) ALT or AST more than 3 times of the normal upper limit, TBIL more than 2 times of the normal upper limit;

8) Serum creatinine levels ≧1.5 mg/dL for males and ≧1.4mg/dL for females or CrCl ≦50 mL/min;

9) Patients with malignant tumors;

10) Patients with mental disorders;

11) History of alcohol abuse or illegal drug abuse;

12) Serious systemic disease not suitable for the study by evaluation of the investigator.

4.2 Subject enrollment

,Participants who have signed the informed consent at Visit 1 will be enrolled and enter the screening period. At this time, investigators will record subjects' identity information and assigned a specific screening number in the *Subject Enrollment Log*.

4.3 Method of Randomization

A central randomization system will be adopted, and the study population will be stratified based on the following: fasting C-peptide (FCP) ≧ 300 pmol/L and < 300 pmol/L; GADA titer ≧0.3 and <0.3 imdex; BMI ≧ 25 kg/m^2 and < 25 kg/m^2. Patients will be randomized 1:1:1 into the following three intervention groups: metformin (and insulin), metformin (and insulin) plus saxagliptin, metformin (and insulin) plus saxagliptin and vitamin D3.

4.4 Procedures for incorrectly enrolled or randomized subjects

Subjects who fail to meet the inclusion or comply with exclusion criteria should not, under any circumstances, be enrolled or receive study medication. There are no exception

to this rule. When patients are incorrectly enrolled, procedures for discontinuation should be followed. When any following situation occurs, i.e. subjects do not meet the inclusion or comply with exclusion criteria, enrolled in error, fail to meet study requirements and incorrectly treated, or when subjects seriously violate the study protocol post initiation, relevant endocrinologists should inform the principle investigator as soon as possible.

Violation of the main inclusion criteria, e.g. non-LADA patients enrolled in error into the study group, relevant results should not be included in the FAS analysis. But subjects of any other incorrect registration or randomization can be included in the FAS analysis. At the same time, investigators will provide subjects with guidance on blood glucose control.

5. INVESTIGATIONAL DRUGS

5.1 Features of investigational product(s)

Investigational drug	Dosage form and strength	Manufacturer
Saxagliptin	5mg, plain, yellow, biconvex, round, film coated tablet	AstraZeneca Pharmaceutical Co., Ltd
vitamin D3	400IU, clear, yellow to orange, capsule	Sinopharm Xingsha Pharmaceuticals (Xiamen) Co., Ltd.

5.2 Treatment regimens

Basic Medication:

First-line drug: Metformin, taken orally

- Daily dosage 1.5g (fluctuate around 1-1.7g/d according to the patients' condition)
- The dosage form, dosage, and frequency of metformin are decided by the investigator

Second-line drug: Insulin, injected subcutaneously

- Daily dosage personalized

- The brand, dosage form, primary dosage, and frequency of insulin are decided by the investigator
- Advice towards the initiation of insulin: from the beginning of the screening stage, if the subject has a level of HbA1c ≧ 7%, the investigator can decide whether or not to initiate insulin treatment (on the basis of continuing metformin), in order to better control the patient's blood glucose level (HbA1c≤7%)
- General advice towards the adjustment of insulin dosage:

➢ The investigator should adjust the dosage and dosage form and decide whether or not to cease the use of insulin according to the patient's blood glucose level, physical condition, and the investigator's clinical experience;

➢ First achieve the glycemic control at an average level and then adjust towards the ideal level, until the patient's blood glucose has reached standard or until the investigator thinks the patient's glucose level is best controlled

➢ First avoid hypoglycemia and then control hyperglycemia.

Investigational Medication:

Saxagliptin: 5mg per pill, orally taken, 1 pill per time, once a day, taken when randomization begins

Vitamin D3: 400IU per pill, orally taken, 5 pills per time, once a day, taken after randomization

Investigational drugs should be taken at approximately the same time of a day during the study, withdrawing saxagliptin 3 days prior to Mixed-meal tolerance test (MMTT).

5.3 Packaging, labeling and storage of the investigational drugs

Packaging of investigational drugs:

Saxagliptin and vitamin D3 will all be provided in its original packaging.

Labelling of investigational drugs:

Drug package of every visit period will have the label indicating "limited to the use of clinical research”.

Study drug storage:

All investigational drugs will be kept in a secure place under appropriate storage conditions (in accordance with the drug instruction). All investigational drugs will be stored under room temperature (below 30°C).

5.4 Compliance Evaluation

The administration of all study drugs (including basic and investigational medication) should be recorded in the appropriate sections of the CRF.

Standard for compliant patient: 80% ≤actual dose taken/prescribed dose*100%≤120%

Standard for non-compliant patient: actual dose taken/prescribed dose*100%≤80% or actual dose taken/prescribed dose*100%≧120%.

Compliance evaluation of basic medication:

Compliance evaluation of metformin: actual dose taken/prescribed dose*100%

Compliance evaluation of insulin: assessed by the investigator according to the dosage form, dosage, and frequency of the insulin injection.

Compliance evaluation of investigational medication:

Evaluation of compliance should be based on the returned tablet count at each visit. The subjects will be asked to return all untaken investigational products and the empty packages of the taken medication.

Compliance evaluation of Saxagliptin: actual dose taken/prescribed dose*100%

Compliance evaluation of vitamin D3: actual dose taken/prescribed dose*100%

Participants judged to be non-compliant can continue the study if the participants understand the importance of taking their study medication as prescribed, and are willing

to comply according to prescription. If the subject continuously fail to comply, the investigator can decide whether or not to terminate participants from this study.

5.5 Prohibition of drugs

Other hypoglycaemic drugs, including sulfonylureas, glinides, alpha-glucosidase inhibitors, thiazolidinediones, GLP-1 agonists, and other DPP-IV inhibitors should not be used throughout the trial.

5.6 Permitted combination medication

Other medication which is considered necessary for the subject's safety and well-being and also beyond the exclusion criteria may be given carefully under the guidance of the investigator(s).

During the course of study, less than twice of systemic corticosteroid treatment with the duration less than a week is allowed if needed due to subject's condition, .

Try best to maintain the dose stability of vitamin D3 and calcium in health supplements and milk during the study as well as record the sunlight exposure time.

When taking strong cytochrome P450 3A4/5 (CYP450 3A4/5) inhibitors(e.g. Ketoconazole, atazanavir, clarithromycin, indinavir, itraconazole, nefazodone, nelfinavir, ritonavir, saquinavir, and telithromycin, etc), reduce the amount of saxagliptin to 2.5mg/d.

6. STUDY PLAN

6.1 Visit schedule

Screening (Visit 1): Screen for patients who were diagnosed with LADA in the 2013BAI09B12 study.

After screening, all subjects who have met the criteria will be enrolled randomly into one of the three treatment groups at the second visit, receiving a 104-week intervention treatment. Follow-up visits will be carried out via telephone interview or outpatient visit (Figure 1 and Table 1).

1st year (Visit 1 - Visit 14) 8 outpatient visits: for the first three months after randomization, outpatient visit is scheduled every month; for the next 9 months, outpatient visit is scheduled every three months; 6 telephone follow-up.

2nd year (Visit 15 - Visit 26) two outpatient visits at 6 month interval; 10 telephone follow-up.

Subjects should keep account of the dose of daily medication, the blood sugar of every SMBG, combination medication and adverse events in the patient diary to ensure their compliance. In addition, to better control blood glucose or in the case of safety issues, the subject may visit their medical center at any time beyond the schedule.

6.2 Measurments during the study:

1) Islet β-cell function indicators: C-peptide level of mixed-meal tolerance test at 0, 60, and 120 minutes;

2) Metabolic control indicators: blood glucose (0, 60, 120 minutes), HbA1c, total cholesterol (TC), low density lipoprotein-cholesterol (LDL-C), high density lipoprotein-cholesterol (HDL-C), Triglyceride (TG);

3) Immunological indicators: GAD-Ab;

4) Chronic complications indicators: electrocardiogram (ECG), Color Doppler Ultrasound of carotid artery, vertebral artery, and lower extremities arteries,

fundus examination and photography, urinary albumin-to-creatinine ratio (UACR), renal function (calculate the estimated glomerular filtration rate, eGFR), electroneuromyography, bone mineral density;

5) Drug safety assessment indicators: complete blood count, aspartate aminotransferase (AST), alanine transaminase (ALT), total bilirubin (TBIL), direct bilirubin (DBIL), serum albumin (ALB), Blood Urea Nitrogen (BUN), creatinine (Cr), uric acid (UA), blood electrolyte, serum 25(OH)D, 24-hour urinary calcium and phosphate, B-ultrasound of the urinary system, and urine pregnancy test;

6) Gene analysis: HLA-DQ, -DR, and vitamin D receptor (VDR) gene polymorphism and immune repertoire polymorphism.

6.3 Time of procedure:

Estimated date of first subject enrolled: January 2016

Estimated date of last subject enrolled: April 2017

Estimated date of last subject completed: May 2019

Estimated date of study report finalized: December 2019

TABLE 1. STUDY PLAN FLOW TABLE

Visit	1	2	3	4	5	6	7	8	9	10	11	12	13	14	15	16	17	18	19	20	21	22	23	24	25	26 [1]
Week	-6	0	4	8	12	16	20	26	30	34	38	42	46	52	56	60	64	68	72	78	82	86	90	94	98	104
Window (week)		0	±1	±1	±1	±1	±1	±1	±1	±1	±1	±1	±1	±1	±1	±1	±1	±1	±1	±1	±1	±1	±1	±1	±1	±1
Study period	Screening	Randomization	Intervention																							
**Follow-up mode**	outpatient	outpatient	outpatient	outpatient	outpatient	phone	phone	outpatient	phone	phone	outpatient	phone	phone	outpatient	phone	phone	phone	phone	phone	outpatient	phone	phone	phone	phone	phone	outpatient
**Written informed consent**	X																									
**Inclusion/Exclusion criteria**		X																								
**Discontinue or not**			X	X	X	X	X	X	X	X	X	X	X	X	X	X	X	X	X	X	X	X	X	X	X	X
**Medical history**	X																									
**Physical examination**	X	X	X	X	X			X			X			X						X						X
**Distribution/collection of patient log**	X	X	X	X	X			X			X			X						X						X
**Adjustment of basic medication[2]**	X	X	X	X	X	X	X	X	X	X	X	X	X	X	X	X	X	X	X	X	X	X	X	X	X	X
**Counting/retrieval of medication[3]**			X	X	X			X			X			X						X						X
**Distribution of medication [3]**		X	X	X	X			X			X			X						X						
**Past/concomitant medications**	X	X	X	X	X	X	X	X	X	X	X	X	X	X	X	X	X	X	X	X	X	X	X	X	X	X
**Compliance evaluation**		X	X	X	X			X			X			X						X						X

**Efficacy evaluation**																										
(0,60,120min)																										
C-peptide* (0,60,120min)	*							*						*						*						*
Blood glucose	X							X						X						X						X
HbA1c	X				X			X						X						X						X
Blood glucose self monitoring and recording	X	X	X	X	X	X	X	X	X	X	X	X	X	X	X	X	X	X	X	X	X	X	X	X	X	X
**Safety Assessment**																										
25(OH)D*	*		*		*			*						*						*						*
Blood routine	X		X		X			X						X						X						X
Blood biochemical test[4]	X		X		X			X						X						X						X
Blood electrolyte	X		X		X			X						X						X						X
Urine pregnancy test[5]	X		X		X			X						X						X						X
24-hour urine calcium and phosphate	X		X		X			X						X						X						X
12-lead ECG	X							X						X						X						X
Urinary system BUS	X		X		X			X						X						X						X
Adverse event		X	X	X	X	X	X	X	X	X	X	X	X	X	X	X	X	X	X	X	X	X	X	X	X	X
**Other Assessment**																										
GADA*	*													*												*
Gene analysis*	*		*		*			*						*						*						*
Complications[6]	X													X												X

*, Centralized test; 1.The contents of the discontinuation visit are consistent with that of visit 26; 2. Metformin (and insulin); 3. Saxagliptin/VitD3 (Group A does not involve any dispensation, check or retrieval of investigational medication; 4.Blood biochemical test include: Lipid (TC, LDL-C, HDL-C, and TG), liver function (AST, ALT, TBIL, DBIL, and ALB), renal function (BUN, Cr, andUA); 5.Apply to women at child-bearing period; 6.Tests for complications include: ① macroangiophathy: 1) Color Doppler Ultrasound of carotid artery, vertebral artery, and lower extremities arteries, 2) ECG; ② microangiopathy: 1) retinopathy: fundus examination and photography, 2) diabetic nephropathy: a.UACR, b. calculate eGFR; ③ peripheral neuropathy: 1) electroneuromyography, 2) physical examination using the tools in the diabetic neuropathy screening kit; ④ osteoporosis: bone mineral density. (Will accept Color Doppler Ultrasound of artery, fundus examination and photography, and electroneuromyography results from Grade A Tertiary Hospitals within 6 months prior to screening; will accept BMD test results from Grade A Tertiary Hospitals within 12 months of randomization).

6.4 Visiting time

Screening

6.4.1 Visit 1 (Week -6 - Week 0) 【outpatient visit】

- Sign written informed consent.
- Medical history
- Physical examination
- Distribution of patient diary and glucose meter
- Initiation or adjustment of basic medication[2]
- Record of past/ concomitant medication (record medication used within 6 months before the screening date)
- MMTT test 0, 60, 120 min blood glucose (conduct at each study site); 0, 60, 120 min C-peptide (deliver to the central laboratory)
- GADA, 25(OH)D test, gene analysis (deliver to the central laboratory)
- HbA1C, blood routine test, blood biochemical[4], blood electrolyte, urine pregnancy test[5], 24-hour urine calcium and phosphate
- 12-ECG
- BUS of urinary system
- Tests for complications[6]

Randomization

6.4.2 Visit 2 (Week 0) 【outpatient visit】

- Verify inclusion/exclusion criteria
- Physical examination

- Collection/distribution of patient diary
- Adjustment of basic medication
- Dispensation of investigational medication
- Record past/ concomitant comitant medication
- Evaluate compliance by the investigator according to the subject's basic medication and self-monitored blood glucose level
- Record adverse events

Intervention

6.4.3 Visit 3 (Week 4 ±1week) 【outpatient visit】

- Check for discontinuation of the study
- Physical examination
- Collection/distribution of patient diary
- Adjustment of basic medication[2]
- Counting/retrieval of investigational medication[3]
- Dispensation of investigational medication[3]
- Record past/ concomitant medication
- Evaluation of compliance
- 25(OH)D test, gene analysis (deliver to the central laboratory)
- blood routine test, blood biochemical[4], blood electrolyte, urine pregnancy test[5], 24-hour urine calcium and phosphate
- BUS of urinary system

- Record adverse events

6.4.4 Visit 4 (Week 8 ±1week) 【outpatient visit】

- Check for discontinuation of the study
- Physical examination
- Collection/distribution of patient diary
- Adjustment of basic medication[2]
- Counting/retrieval of investigational medication[3]
- Dispensation of investigational medication[3]
- Record past/ concomitant medication
- Evaluation of compliance
- Record adverse events

6.4.5 Visit 5 (Week 12 ±1week) 【outpatient visit】

- Check for discontinuation of the study
- Physical examination
- Collection/distribution of patient diary
- Adjustment of basic medication[2]
- Counting/retrieval of investigational medication[3]
- Dispensation of investigational medication[3]
- Record past/ concomitant medication
- Evaluation of compliance
- 25(OH)D test, gene analysis (deliver to the central laboratory)

- HbA1c, blood routine test, blood biochemical[4], blood electrolyte, urine pregnancy test[5], 24-hour urine calcium and phosphate
- BUS of urinary system
- Record adverse events

6.4.6 Visit 6 (Week 16 ±1week); Visit 7 (Week 20 ±1week)【phone visit】

- Check for discontinuation of the study
- Adjustment of basic medication
- Record past/ concomitant medication
- Supervise the recording of patient diary (including blood glucose self-monitoring, etc.)
- Record adverse events

6.4.7 Visit 8 (Week 26 ±1week) 【outpatient visit】

- Check for discontinuation of the study
- Physical examination
- Collection/distribution of patient diary
- Adjustment of basic medication[2]
- Counting/retrieval of investigational medication[3]
- Dispensation of investigational medication[3]
- Record past/ concomitant medication
- Evaluation of compliance
- MMTT test 0, 60, 120 min blood glucose (conduct at each study site); 0, 60, 120 min C-peptide (deliver to central laboratory)
- 25(OH)D test, gene analysis (deliver to the central laboratory)

- HbA1c, blood routine test, blood biochemical[4], blood electrolyte, urine pregnancy test[5], 24-hour urine calcium and phosphate
- 12-lead ECG
- BUS of urinary system
- Record adverse events

6.4.8 Visit 9 (Week 30 ±1week); Visit 10 (Week 34 ±1week) 【phone visit】

- Check for discontinuation of the study
- Adjustment of basic medication
- Record past/ concomitant medication
- Supervise the recording of patient diary (including blood glucose self-monitoring, etc.)
- Record adverse events

6.4.9 Visit 11 (Week 38 ±1week) 【outpatient visit】

- Check for discontinuation of the study
- Physical examination
- Collection/distribution of patient diary
- Adjustment of basic medication[2]
- Counting/retrieval of investigational medication[3]
- Dispensation of investigational medication[3]
- Record past/ concomitant medication
- Evaluation of compliance
- Record adverse events

6.4.10 Visit 12 (Week 42 ±1week); Visit 13 (Week 46 ±1week) 【phone visit】

- Check for discontinuation of the study
- Adjustment of basic medication
- Record past/ concomitant medication
- Supervise the recording of patient diary (including blood glucose self-monitoring, etc.)
- Record adverse events

6.4.11 Visit 14 (Week 52 ±1week) 【outpatient visit】

- Check for discontinuation of the study
- Physical examination
- Collection/distribution of patient diary
- Adjustment of basic medication[2]
- Counting/retrieval of investigational medication[3]
- Dispensation of investigational medication[3]
- Record past/ concomitant medication
- Evaluation of compliance
- MMTT test 0, 60, 120 min blood glucose (conduct at each study site); 0, 60, 120 min C-peptide (deliver to central laboratory)
- GADA, 25(OH)D test, gene analysis (deliver to the central laboratory)
- HbA1c, blood routine test, blood biochemical[4], blood electrolyte, urine pregnancy test[5], 24-hour urine calcium and phosphate
- 12-lead ECG

- BUS of urinary system
- Tests for complications[6]
- Record adverse events

6.4.12 Visit 15 (Week 56 ±1week); Visit 16 (Week 60 ±1week); Visit 17 (Week 64 ±1week); Visit 18 (Week 68 ±1week); Visit 19 (Week 72 ±1week) 【phone visit】

- Check for discontinuation of the study
- Adjustment of basic medication
- Record past/ concomitant medication
- Supervise the recording of patient diary (including blood glucose self-monitoring, etc.)
- Record adverse events

6.4.13 Visit 20 (Week 78 ±1week) 【outpatient visit】

- Check for discontinuation of the study
- Physical examination
- Collection/distribution of patient diary
- Adjustment of basic medication[2]
- Counting/retrieval of investigational medication[3]
- Dispensation of investigational medication[3]
- Record past/ concomitant medication
- Evaluation of compliance
- MMTT test 0, 60, 120 min blood glucose (conduct at each study site); 0, 60, 120 min C-peptide (deliver to central laboratory)

- 25(OH)D test, gene analysis (deliver to the central laboratory)
- HbA1c, blood routine test, blood biochemical[4], blood electrolyte, urine pregnancy test[5], 24-hour urine calcium and phosphate
- 12-lead ECG
- BUS of urinary system
- Record adverse events

6.4.14 Visit 21 (Week 82 ±1week); Visit 22 (Week 86 ±1week); Visit 23 (Week 90 ±1week); Visit 24 (Week 94 ±1week); Visit 25 (Week 98 ±1week) 【phone visit】

- Check for discontinuation of the study
- Adjustment of basic medication
- Record past/ concomitant medication
- Supervise the recording of patient diary (including blood glucose self-monitoring, etc.)
- Record adverse events

6.4.15 Visit 26 (Week 104 ±1week)/Discontinuation Visit 【outpatient visit】

- Physical examination
- Collection/distribution of patient diary
- Adjustment of basic medication[2]
- Counting/retrieval of investigational medication[3]
- Record past/ concomitant medication

- Evaluation of compliance
- MMTT test 0, 60, 120 min blood glucose (conduct at each study site); 0, 60, 120 min C-peptide (deliver to the central laboratory)
- GADA, 25(OH)D test, gene analysis (deliver to the central laboratory)
- HbA1C, blood routine test, blood biochemical[4], blood electrolyte, urine pregnancy test[5], 24-hour urine calcium and phosphate
- 12-lead ECG
- BUS of urinary system
- Tests for complications[6]
- Record adverse events

Note: 1.The contents of the discontinuation visit are consistent with that of visit 26; 2.Metformin (and insulin); 3.Saxagliptin/VitD3 (Group A does not involve any dispensation, check or retrieval of investigational medication; 4. Blood biochemical test include: Lipid (TC, LDL-C, HDL-C, and TG), liver function (AST, ALT, TBIL, DBIL, and ALB), renal function (BUN, Cr, andUA); 5. Apply to women at child-bearing period; 6.Tests for complications include: ① macroangiopathy: 1) Color Doppler Ultrasound of carotid artery, vertebral artery, and lower extremities arteries, 2) ECG; ② microangiopathy: 1) retinopathy: fundus examination and photography, 2) diabetic nephropathy: a.UACR, b. calculate eGFR; ③ peripheral neuropathy: 1) electroneuromyography, 2) physical examination using the tools in the diabetic neuropathy screening kit; ④ osteoporosis: bone mineral density. (Will accept Color Doppler Ultrasound of artery, fundus examination and photography and electroneuromyography reports from Grade A Tertiary Hospitals within 6 months prior to screening; will accept BMD test results from Grade A Tertiary Hospitals within 12 months of randomization).

6.5 Screening Failure

Participants who fail the screening refer to those who have signed the informed consent, but do not enter the randomization.

Subjects dropped out after randomization (refer to 6.6 Discontinuation of Study) will not be replaced by new subjects, and the subjects' numbers could not be used again. Subjects cannot be enrolled into the study again once dropped out.

Subjects who have signed the consent form but failed in the screening period before randomization, are allowed to go through screening again if inclusion or exclusion criteria has changed. In this case, subjects have to sign the new consent forms and be assigned to new randomization numbers.

6.6 Discontinuation of Study

Patients may be discontinued from the study if any of the following situations occur:

- Voluntary discontinuation by the subject, revoking the informed consent;
- Safety reasons as judged by the investigator;
- Severe non-compliance to protocol as judged by the investigator;
- Incorrect enrollment, i.e. the patient does not meet the required inclusion/exclusion criteria;
- Patient lost to follow-up (as defined by, unable to reach the patient after 3 valid phone calls, fax or email, all contact should be documented in the patient's medical records);
- Pregnancy;

Investigators should record the reasons of discontinuation or drop-out subjects. Subjects need to go through relevant process and inspection of discontinuation (identical to the 26st follow-up). The random coding and research drugs of these subjects cannot be passed on to other subjects.

7. STUDY ASSESSMENTS

The investigator must ensure that all data are accurately, completely, and timely recorded in the outpatient medical record (or study medical record) and EDC according to the research protocol.

7.1 Efficacy assessments

- C-peptide: they were tested in a central laboratory. Detection was carried out by chemiluminescence using a commercially available kit (Adiva Centaur System, Siemens, Germany). The intra- and inter-assay variation coefficients were 3.7-4.1% and 1.0-3.3% respectively. Fasting serum C-peptide, peak C-peptide (60 minutes) and the area under curve of the 120-minute C-peptide were measured using a mixed meal tolerance test (MMTT, as follows). Detection is performed at V1, V8, V14, V20, and V26.

- HbA1c: it was tested in local medical center laboratories independently. The measurements are performed at V1, V5, V8, V14, V20, and V26.

- MMTT: participants will be provided with instant noodles with the net weight of 75g, and without any condiment oil. Timing of the 1-hour and 2-hour postprandial tests starts from the beginning of the meal. Participants will be required to finish the meal in 15 minutes once started. Three days before the MMTT, the participants must be reminded to assure at least 150 grams of carbohydrate intake every day Before visiting, the participants must have fasted for at least 8 hours and abstained from tobacco, alcohol and caffeine for at least 24 hours. Confirm that the patient did not take saxagliptin 3 days before visit, and did not take the insulin injection in the morning of the visit date.

7.2 Safety assessments

The following will be measured for the safety of the study

Monitor and record all adverse events (AEs) (see study protocol 8.1) and severe adverse events (SAEs) (see study protocol 8.2), combined medications, hematology, blood biochemical and urine tests, ECG, as well as vital signs, weight, and so on.

7.2.1 Laboratory tests

The sponsor will assign the central laboratory to carry out the safety assessment: test of 25(OH)D. Other clinical lab tests will be carried out at laboratories of each research center.

Central laboratory test items:

25(OH)D: using vitamin D detective kit from SIEMENS (chemiluminescence method), Detection was performed at V1, V3, V5, V8, V14, V20 and V26.

Individual research center laboratory test items:

type		parameter
Blood	Blood routine	Blood cell count (RBC, hemoglobin, mean corpuscular volume, leukocytes) differential blood count (neutrophils, lymphocytes, monocytes, eosinophils, basophils) and platelets.
	Liver and renal function	AST, ALT, TBIL, DBIL, ALB, BUN, Cr, UA
	Lipid	TC, HDL-C, LDL-C, TG
	Electrolyte	Calcium, Phosphorus, Sodium, Potassium, Chloride, Magnesium
	Others	HbA1c, Blood glucose
Urine	Pregnancy test	Urine pregnancy test
	Urine test	24-hour urine calcium and phosphate; UACR

Abbreviations: AST – aspertate transaminase, ALT – alanine transaminase, TBIL – total bilirubin, DBIL – direct bilirubin, ALB-serum albumin, BUN – blood urea nitrogen, Cr – creatinine, UA – uric acid, TC – total cholesterol, HDL-C – high density lipoprotein cholesterol, LDL-C – low density lipoprotein cholesterol, TG – triglyceride; UACR – urinary albumin-to-creatinine ratio.

Any laboratory result abnormality might meet the qualitative criteria for AE in this study (reference to 8.1.1). In these cases, all AEs related to abnormal lab results should be recorded in the CRF.

7.2.2 Physical examination

The physical examination includes the following: height, weight, waist circumference, hip circumference, general appearance, superficial lymph nodes, skin and sclera inspection, skull, eyes, ENT, neck, chest, respiratory, cardiovascular, abdomen, musculoskeletal/extremities, and nervous system (must perform the following examinations using the neurology screening kit: a. ankle reflex; b. tactile test with a 10g nylon thread; c. pallesthesia sensibility via tuning fork; d. needle algesia test; e. thermesthesia sensibility). Baseline data were collected at Visit1. Physical examination items that were found to have changed during subsequent follow-up should be recorded for comparison with baseline.

7.2.3 Pulse and blood pressure

Pulse and blood pressure (BP) should be measured when the patient is quiet and in a sitting position (the patient should have rested comfortably for at least five minutes) and with their right arm outstretched at the same level as the middle part of the sternum. Measurement should be taken under standardized conditions, approximately at the same time of each day, and on the same arm. BP should be measured with the same device and the values be recorded in the original files and in the CRF (the sphygmomanometer should be calibrated regularly according to the manufacturers' instructions). Both systolic blood pressure (SBP), diastolic blood pressure (DBP), and pulse rate should be recorded. Patients with complications of microangiopathy and neurophathy should be tested for autonomic neuropathy, for instance, measurement of postural BP. Postural BP measurement: The patient should lie in supine position for at least 5 minutes before his/her supine BP and pulse could be measured. The cuff should be fastened around his/her right upper arm (be careful not to unite the cuff). Next, the patient should remain standing quietly for 1 minute and then have his/her orthostatic BP and pulse measured (with the same method as measuring a seated BP, where the patient's right arm is outstretched and the cuff is at the same level as the left atrium). The patient should remain standing for 3 more minutes and have his/her BP and pulse measured again. Hypotension could happen immediately or tardively after standing. The condition, where the orthostatic SBP is 20 mmHg lower or DBP is 10 mmHg lower than the supine SBP and DBP respectively, is called postural hypotension.

7.2.4 Body temperature (when suspecting fever)

Body temperature should be measured using a thermometer in degrees Celsius when the patient is quiet, and axillary temperature should be recorded in the CRF. Once the temperature is higher than 38.5 centigrade, the patients should withhold the visit until his or her temperature returned to the normal range (record as an adverse event).

7.2.5 ECG

The 12-lead ECGs should be performed after at least 10 minutes in supine position. The electrodes are to be positioned at the same place for each ECG recording throughout the study. The investigator has to review the ECG and document his interpretation, including

signature and date on the ECG print-out. The original trace and its copies were saved as source data and recorded in the e-CRF according to the investigator's evaluation.

7.2.6 BUS of urinary system

B-Ultrasound examination of the urinary system often includes bilateral kidneys, ureters, bladder (the prostate, for male). Before the examination, the doctor must apply a facial cleanser like coupling agent to the detector, so that the detector could have better contact with skin and the ultrasonic wave could detect into the body. The coupling agent is safe, non-toxic, non-irritating to the skin and could be cleaned with soft paper after the examination. It does not require fasting situation or taking oral intestinal cleaning agent, but the patients are required to drink 300~500 ml water and preferably not urinate within 2~3 hours.

7.3 Other assessments

- GADA: tested in a central laboratory by using radioimmunoassay. The sensitivity and specificity were 82% and 98%, respectively. The assay was sponsored by the Immunology of Diabetes Society (IDS) and has been validated by Islet Autoantibody Standardization Program (IASP) in 2012. Changes of GADA titers will be measured once a year during intervention: at V14 (week 52), and V26 (week 104).

- Genetic testing: tested in a central laboratory. HLA-DQA1, -DQB1, HLA-DRB1, and VDR genotypes will be determined by DNA analysis using polymerase chain reaction (PCR) to amplify exon of DQA1, DQB1, DRB1, and VDR genes followed by standard DNA sequencing-based typing. DNA samples were collected before and after the intervention, and high-throughput sequencing was used to detect changes in immune diversity before and after treatment. Detection is performed on V1, V3, V5, V8, V14, V20 and V26.

- Complications: The complications will be examined according to the conditions of each hospital, which includes: ① macroangiophathy: 1) Color Doppler Ultrasound of carotid artery, vertebral artery, and lower extremities arteries, 2) ECG; ② microangiophathy: 1) retinopathy: fundus examination and photography, 2) diabetic nephropathy: a. UACR, b. calculate eGFR; ③ peripheral neuropathy: 1) electroneuromyography, 2) physical examination using the tools in the neurology screening kit; ④ osteoporosis: bone mineral density. (We will accept Color Doppler Ultrasound of artery, fundus examination and photography, and electroneuromyography results from Grade A Tertiary Hospitals within 6 months prior to screening, and will accept bone mineral density test results from Grade A Tertiary Hospitals within 12 months before the date of randomization).

a）Urinary albumin-to-creatinine ration (UACR): collect 10-15ml of the participant's urine in clean, regular urine collection tube (or small bottle), and correctly label the basic information of the participant. The specimens do not require further process but to be sealed and tested at each hospital. Attention: do not get vaginal secretion or faeces mixed into the urine sample; patients with hematuria caused by urinary infection or other causes should be re-tested after the hematuria is controlled.

b）Calculate the estimated glomerular filtration rate (eGFR): use the CKD-EPI formula, or use the "National Kidney Foundation Calculators" to calculate (GFR caculator website: https://www.kidney.org/professionals/KDOQI/gfr_calculator).

7.4 Collection and processing of biological samples

Refer to Appendix 3: 《*Samples collection, transport and test results report procedures*》

8. SAFETY REPORTING AND MEDICAL MANAGEMENT

The Principal Investigator is responsible for ensuring that all staff involved in the research are familiar with the content of the study.

8.1 Definition of adverse events (AE) and treatment plans

Adverse Event refers to an adverse medical event that occurs after a patient or a participant receives treatment, but does not necessarily have a causal relationship with the treatment. Once an adverse event occurs, detailed records, judgements on severity, timely diagnosis and effective treatments and follow-up of the subjects are required.

8.1.1 Recording of adverse events

Regardless of seriousness or relationship to the investigational drug, all AEs should be recorded on the corresponding page(s) in the CRF from the time the subject signed the informed consent form until the end of the study as defined by the protocol for that patient, Any laboratory tests, vital signs or other abnormalities that are medically relevant and clinically meaningful (causing any type of intervention) should be recorded as an adverse event. Including symptomatic; and/or requiring either corrective treatment or consultation; and/or leading to discontinuation or modification of dosing; and/or fulfilling a seriousness criterion; and/or defined as an AE with pre-specified monitoring with immediate notification.

8.1.2 Intensity of adverse events and management

When filling out the adverse event table, the investigator will use mild, moderate, and severe to describe the intensity of adverse events. To harmonize standards, the intensity of events is ranked as follows.

(1) Mild: The event does not interfere with the normal daily activity of the subject, and there is no need for symptomatic treatment and discontinuation of dosing.

(2) Moderate: The event partly affects the subject’s normal daily activity and requires symptomatic treatment or discontinuation of dosing.

(3) Severe: The event obviously interferes with subject’s normal daily activity and requires emergency treatment or immediate discontinuation of dosing.

8.1.3 Treatment of adverse events

Investigators should determine whether to suspend the clinical trial according to the severity of the event and provide the diagnosis and necessary treatment based on his/her clinical experience. All adverse events should be tracked for investigation, the process and the result of the treatment should be recorded in details until the situation is properly solved or the patient's condition is stable, abnormal test results must be followed until the figures are normal.

The relevant treatment on some particular adverse events, such as severe nausea and vomiting, hypoglycemia, hypercalcemia and vitamin D intoxication should refer to 8.4-8.7.

8.1.4 Follow-up of adverse events

Any AEs are unresolved or recovered at the last visit/study discontinuation or subject withdrawal from study, the subjects are followed up by the investigator for as long as AEs are relieved/ recovered to baseline/no longer worsening or stable/interpretable by investigator (for the worsening of diseases unrelated to the investigational drug, and without relieved/recovered condition since 30 days from the end of visit)/lost to follow-up.

8.2 Definition of serious adverse events (SAEs)

Serious adverse events: in the course of clinical trials, meeting the necessity of hospitalization or prolongation of hospitalization, disability, incapacity of work, life-threatening or death, congenital abnormality and so on, listed as followings:

1) Leading to death
2) life-threatening
3) Requires in-patient hospitalization or prolongation of existing hospitalization
4) lead to persistent or significant disability/incapacity or substantial disruption of the ability to conduct normal life functions

5) Cause congenital abnormality or birth defects, etc.

6) Important medical events (defined as a medical event that may not be immediately life-threatening or result in death or hospitalization but based upon appropriate medical and scientific judgment, that may jeopardize the subject or may require medical intervention to prevent various serious outcomes listed in the definition above).

The following hospitalization does not regard as SAEs, for there is no "adverse event" associated with hospitalization (eg, there's no adverse medical event):

1) Hospitalization for convenience of fulfilling the examination on complications referred in the study protocol;
2) Hospitalization for convenience of routine physical examination;
3) Hospitalized for temporal medical care.
4) Plan to hospitalize before sign Informed Consent (after taking investigational drug, there's no change for the plan to hospitalize).

It is important to distinguish between serious and severe AEs. An AE of severe intensity does not necessarily need to be defined ad a serious adverse event. For example, nausea that persists for several hours may be considered severe nausea, but not a SAE. On the other hand, a stroke that results in only a limited degree of disability may be considered a mild stroke but still would be a SAE.

8.2.1 Reporting of serious adverse events

For any SAEs occurring during study phase, investigators can carry out rescue and put on records according to each hospital's standard operation procedure (SOP) on first aid emergencies. Investigator should report to the sponsor, AstraZeneca Patient Safety Department, Ethics Committee (EC), as well as provincial or municipal drug regulatory agency and China Food and Drug Administration (CFDA) within 24 hours since he/she is aware of it.

Investigators must be responsible for the follow-up of subjects with SAEs regularly. Follow-up reports about SAEs should be reported to the sponsor, AstraZeneca Patient Safety Department and EC within 24 hours.

Contact Detail

Contact	Telephone	Fax	E-mail
Safety Supervision Department of CFDA	010-68313344-1003	010-88363228	-
Registration Department of CFDA	010-68313344-1009	010-88363236	
Sponsor	0731-85367220<br>137-3905-7331	0731-85367220	ncrcmd@163.com
AstraZeneca Patient Safety Department	021-38683551	021-38683551	China.AZDrugSafety@astrazeneca.com

8.3 Causality assessment of (severe) adverse events

The Investigator will assess causal relationship between investigational product and each AE/SAE, as well as the possible association with concomitant medicine using the following 5-level criteria:

Definite: It follows a reasonable temporal sequence from drug administration; it follows a known response pattern to suspected drug; it dispears on cessation of the suspected drug; it can't be caused by the subject's clinical state or other reasons.

Probably: It follows a reasonable temporal sequence from drug administration; it follows a known response pattern to suspected drug; it is obviously alleviated on cessation of the suspected drug; it can't be caused by the subject's clinical state or other reasons.

Possibly: It follows a reasonable temporal sequence from drug administration; it follows a known response pattern to suspected drug; it may be alleviated on cessation of the suspected drug; it may have been caused by the subject's clinical state or other reasons.

Unlikely: It may not follow reasonable temporal sequence from drug administration; it may not follow a known response pattern to suspected drug; it is not relieved on cessation of the suspected drug; it may have been produced by the subject's clinical state or other reasons and it may decrease when disease state improve or other reason removed.

Unrelated: It does not follow reasonable temporal sequence from drug

administration; It does not follow a known response pattern to suspected drug; It may readily have been caused by the subject's clinical state or other reasons and it may be relieved when disease state improve or other reason removed.

For SAEs, investigator will also assess causal relationship (with all medication/study procedures), and discussed with sponsor.

8.4 Treatment on severe nausea and vomiting

If the patient experience persistent or severe nausea and vomiting, investigators should look for possible causes. If there is clinical indication, serum creatinine should be measured, if serum creatinine rises sharply, metformin should be suspended until the recovery of renal function.

8.5 Treatment on hypoglycemia

Hypoglycemia includes asymptomatic and symptomatic hypoglycemia.

- Asymptomatic hypoglycemia: patient shows no discomfort, blood glucose < 3.9mmol/L. Carbohydrates should be taken to correct low blood sugar.
- Symptomatic hypoglycemia: experiencing sweating, palpitations, hunger, restlessness, anxiety, fatigue, irritability, headaches, difficulty in concentrating, lethargy, and visual disturbances, transient sensory or motor deficits, confusion, convulsions or coma, etc., monitoring of blood glucose level < 3.9mmol/L, or, above symptoms will immediately recover after administration of oral carbohydrates, intravenous glucose or glucagon even without obtaining blood glucose testing values,.
- Severe symptomatic hypoglycemia: acute neurological deficit directly caused by hypoglycemia events leads to the unability to self-treatment and the need of the assistance of others. The following characteristics will be shown: (1) blood sugar levels < 2.0mmol/L when experiencing the above symptoms; (2) without obtaining blood glucose level, but administration of oral carbohydrate, intravenous glucose or glucagon can immediately ameliorate symptoms.

Please note that "the need of the assistance of others" means the patient is unable of self-treatment. The situation that assistance is unnecessary, but only due to goodwill, does not meet the standard of "the need of the assistance of others".

When hypoglycemia meets the standards of serious adverse events, it should be reported as a serious adverse event. All convulsions, loss of consciousness or coma should be reported as a serious adverse event.

8.6 Treat on hypercalcemia

Clinical manifestations of hypercalcemia are associated with the magnitude and speed of the increase of serum calcium. Mild hypercalcemia may be asymptomatic. symptoms include anorexia, nausea, vomiting, constipation, fatigue, muscle fatigue, reduce of muscle tension, polydipsia, polyuria; drowsiness, confusion, and even coma. ECG may show shortening of Q-T interval, the change of ST-T, atrioventricular block and hypokalemic u waves. If not treated timely, it may result in fatal arrhythmia. Long duration of hypercalcemia can lead to calcium deposition within various tissue and organ, such as the conjunctiva, periarticular and kidney stones. It should be noted that serum albumin have impact on calcium concentration. Elevated serum calcium levels can be categorized into mild, moderate and severe.

- Mild hypercalcemia: total serum calcium values above the upper reference limit, but < 3mmol/L;
- Moderate hypercalcemia: total serum calcium values 3-3.5mmol/L;
- Severe hypercalcemia: total serum calcium > 3.5mmol/L, while causing a series of serious clinical manifestations, namely hypercalcemia crisis.

The most common causes of hypercalcemia are primary hyperparathyroidism and malignant tumors, other reasons include: drugs: such as vitamin D, vitamin A, thiazide diuretics, lithium, theophylline, estrogen and anti-estrogen hormones; endocrine diseases, sarcoidosis and other granulomatous diseases, long-term immobilization, familial hypocalcinuric hypercalcemia and milk alkali syndrome. Once the patient's serum calcium is high:

- Confirm the existence of hypercalcemia, measure of serum calcium should be repeated to rule out laboratory error and prolonged tourniquet lashing or other human factors; be aware whether the patient is dehydrated or rise of plasma protein concentration.

- Once hypercalcemia is confirmed, the cause should be identified: researchers can determine whether to simultaneously monitor PTH, 25(OH)D, renal function, urinary calcium/creatinine clearance rate and alkaline phosphatase based on the patient's condition, and seek for other possible causes; if the patient is in the vitamin D3 treatment group, vitamin D3 therapy should be suspended.

- Treatment of hypercalcemia depends on calcium levels and clinical symptoms. In aspect of mild hypercalcemia, generally no active measures are taken to control blood calcium in patients without clinical symptoms; for symptomatic moderate and severe hypercalcemia patients, immediate treatment may be required according to the cause; when calcium > 3.5 mmol/L, regardless of the presence or absence of clinical symptoms, immediate and effective measures are required: dilatation, promote urinary calcium excretion, inhibit bone absorption, glucocorticoids, and even dialysis to lower blood calcium [26].

When the cause and manifestation of hypercalcemia meet the standards of serious adverse events, it should be reported as a serious adverse event.

8.7 Treatment on vitamin D intoxication

Vitamin D intoxication has no particular manifestation, similar to hypercalcemia. Early phase of acute intoxication first presents with increased serum 25-OHD and calcium, change of epiphysis appearring later. Abnormal X-rays can assist diagnosis, but negative results cannot rule out the possibility of vitamin D intoxication.

In the “Application Guideline for Vitamin D and Bone Health in Adult Chinese”, it refers American guideline that it is considered as potential vitamin D intoxication if 25(OH)D > 500nmol/L (200ng/ml) continuously. However, the actual incidence of vitamin D intoxication is low and it happens rarely in accidental overdose or inappropriate treatment with large dose.

In this study, the daily dose of vitamin D should be reduced to 800IU, if 25(OH)D>250nmol/L. When serum 25(OH)D>500nmol/L (200ng/mL), study should be suspended, withdrawal vitamin D and calcium, avoid sunlight, apply low calcium diet. Adrenal hormones can inhibit the intestinal absorption of calcium, and antagonize VitD. its impact on blood glucose should be noted.

When vitamin D intoxication meets the standards of serious adverse event, it should be reported as serious adverse event.

8.8 Overdose

An overdose (accidental or intentional) with the investigational drugs is an event suspected by the investigator or spontaneously notified by the patient and defined as at least twice of the intended dose within the intended therapeutic interval (e.g. within one calendar day)-. If an overdose on a AZ study drug occurs in the course of the study, then investigators or other site personnel inform sponsor immediately when he or she becomes aware of it.

An overdose with associated AEs should be recorded as the AE diagnosis/symptoms on the relevant AE modules in the CRF and also on the Overdose CRF module. An overdose without associated symptoms only needs to be reported on the Overdose CRF module.

8.9 Pregnancy

Before signing the informed consent, investigators should notify childbearing age participants of the importance of contraception throughout the study and the potential risks of accidental pregnancy. The subject must sign the informed consent to claim that the investigator has discussed the risk factors and outcomes with her.

If a subject becomes pregnant during the course of the study, investigational product should be discontinued immediately and the procedure of study discontinuation of the subject should be finished. Pregnancy itself is not regarded as an adverse event unless there is a suspicion that the investigational product under study may have interfered with the effectiveness of a contraceptive medication. Congenital abnormalities/birth defects and spontaneous miscarriages should be reported and handled as SAEs. Elective abortions without complications should not be handled as AEs. The outcome of all pregnancies (spontaneous miscarriage, elective termination, ectopic pregnancy, normal birth or congenital abnormality) should be followed up and documented even even the subject was discontinued from the study.

If any pregnancy occurs in the course of the study, then investigators or other site personnel inform sponsor within one day.

The same timelines apply when outcome information is available.

9. STATISTICAL ANALYSIS

All data are analyzed by SAS 9.3 software (SAS Institute Inc.,Cary,NC,USA).

9.1 Statistical considerations

Statistical analysis will be performed by selected biostatistics professionals. A comprehensive Statistical Analysis Plan (SAP) will be prepared before database locked. Exploratory analysis is allowed to make changes to adapt to unexpected issues during the study.

9.2 Sample size estimation

The sample size calculations were performed based on the primary variable "absolute change in fasting C-peptide from baseline to 104-week".

Because there is no study for the effect of Saxagliptin combined with vitamin D3 on β-cell function of diabetes patients, the sample size calculations in this study is based on early published literature comparing the effect of sitagliptin combining insulin and insulin therapy alone on β-cell function in LADA patients with the course of diabetes less than1 year. Our study hypothesis is that saxagliptin will improve the protective effect on β-cell function in LADA treated with metformin (and insulin) than those with metformin (and insulin) alone, and saxagliptin combined with vitamin D will also further improve protective effect on β cell function in LADA treated with metformin (and insulin) than those with metformin (and insulin) alone. The means of effect of insulin treatment group and sitagliptin combining insulin group on fasting C-peptide (extent of fasting C-peptide changing from baseline to 1 year later) which is the indication of β-cell function are 149.2, 108.4 individually, the standard deviations are 84.5, 52.8 individually. As twice test involved, set two-sided α=0.025 as significance level, and set β=0.1 that means the power of test is 90%. According to the formula used to compare the means of two-sample:

$$n = 2\left[\frac{(\mu_\alpha + \mu_\beta)}{\delta/\sigma}\right]^2 + \frac{1}{4}\mu_\alpha^2$$

Calculating with the formula and above values, we can get the sample size n=76.

According to calculation with the drop-out rate which is approximate 20%, 95 patients per group are needed. We set 100 cases per group, 300 cases in total.

9.3 Definitions of analysis sets

The primary efficacy analysis population will be based on the Full Analysis Set (FAS) and the Per-Protocol Analysis Set (PP). The secondary analysis population and the safety analysis population will be based on the Per-Protocol Analysis Set (PP) and Safety Analysis Set (SS), respectively.

9.3.1 Efficacy analysis set

Intention-to-treat (ITT) analysis

the results of an experiment is based on the initial treatment assignment but not on the treatment eventually received. ITT analysis is intended to avoid various misleading artifacts that can arise in intervention research such as non-random attrition of participants from the study or crossover. ITT is also simpler than other forms of study design and analysis because it does not require observation of compliance status for units assigned to different treatments or comprise the incorporation of compliance into the analysis.

Full analysis set (FAS)

The Full analysis set will include all randomized subjects, including those who took at least 1 dose after randomization, and had at least 1 baseline and post-baseline efficacy data assessments. Subjects on FAS will be analyzed by the treatment group they were randomized to. The Full analysis set will be the primary set for efficacy analysis in the study.

Per protocol (PP) analysis set

The PP analysis set is a subset of the Full analysis set that includes subjects who do not have significant protocol deviations that affect the study outcome. These exclusions from the PP analysis set will include but not be limited to the patients who took prohibited concomitant medications and major protocol deviations or violations. The exclusions from the PP analysis set will be determined prior to database lock on data review meeting.

9.3.2 Safety analysis set

Safety analysis Set (SS) is a subset of all patients enrolled set including patients who take at least one Investigational Product (IP) dose and have any safety records that can be found in the study database. Analysis will only use values that are actually measured.

9.4 Outcome measures for analysis

The statistical description includes mean, standard deviation, median, quartile and maximum value and minimum value of continuous variables, and frequency and percentile of categorical variables. All data will be examined for outliers and missing values. Some variables will be transformed. Analysis will be based on the data distribution and research issues. Continuous variables will be analyzed with ANOVA for randomized block design, paired t-test and rank sum test to compare the changes from baseline within the same group. ANOVA and rank sum test will be used to examine the differences between groups for continuous variables. Categorical variables will be analyzed with Pearson chi-square test, CMH chi-square test or Fisher probabilities to compare the differences between groups. A P value of <0.05 is considered to be statistically significant except for extra specification.

Demographic and baseline characteristics: Frequency distribution and summarized statistical data of demographic and baseline variables will be analyzed individually according to different treatment groups in efficacy analysis.

9.4.1 Analysis of the primary variable(s)

The primary endpoints in the study are absolute fasting C-peptide changes between saxagliptin + metformin (and insulin) group vs. metformin (and insulin) group, and vitamin D + saxagliptin + metformin (and insulin) group vs. metformin (and insulin) group from baseline after 104 weeks of treatment. The primary endpoint will be analyzed utilizing mixed model repeated measures (MMRM) on full analysis set (FAS). Within the framework of MMRM, point estimations and 2-sided 95% CIs will be presented at each visit for the mean change within each treatment group as well as the difference of the mean change between treatment groups. The following hypothesis will be tested to establish superiority:

Hypothesis Testing in Superiority Trial:

Superiority trials are designed to show a treatment effect between saxagliptin (combined with vitamin D3) + metformin (and insulin) group and metformin (and insulin) alone group . Treatment with saxagliptin (combined with vitamin D3) + metformin (and insulin) is supposed to be superior. This superiority trial can be expressed by two hypotheses:

- Null hypothesis H0: μS-μI = 0, which states that there is no difference between saxagliptin (and vitamin D3) + metformin (and insulin) group and metformin (and insulin) alone group in terms of outcome variables;
- Alternate hypothesis Ha: μS-μI≠ 0, which states that there is a difference.

The type I error rate for each hypothesis test is adjustied to be 0.025 considering saxagliptin + metformin (and insulin) group vs. metformin (and insulin) group and saxagliptin + vitamin D3 + metformin (and insulin) group vs. metformin (and insulin) group involved.

To determine whether saxagliptin was effective, the confidence intervals for the two groups should be constructed. If the two-tailed upper limitation of 97.5% CI is less than 0, then null hypothesis H0 is rejected and a "win" declared if the absolute change of fasting C-peptide is 100 pmol/L or less in Group B in the 104 weeks. A sensitivity analysis will also be performed on the primary endpoint with last observation carried forward (LOCF) for missing values by analysis of covariance (ANCOVA). The effect of "drop-outs" (subject withdrawals), covariates (age, gender, physiologic status), and protocol violations will be analyzed by statistical models.

9.4.2 Analysis of the secondary variable(s)

The secondary endpoints including other parameters of β-cell function, for example C–peptide changes during MMTT. Glycaemic control parameters including HbA1c, FPG and PPG. Insulin-sparing effect is the difference of daily insulin dosage. One-way ANOVA will be used to examine the baseline data and the changes in AUC during MMTT, FPG, PPG, HbA1c and insulin dose between groups. The changes of secondary variables described above from the baseline within the same group and between groups will be analyzed with repeated measurement analysis of variance. The frequency counts

and percentages will be summarized on proportion of patients achieving β-cell function protection and glycaemic control.

9.4.3 Analysis method for safety endpoints:

The incidence of adverse events (AEs) and apparent abnormal central laboratory results will be summarized according to different treatment groups. All severe adverse events and AEs that cause discontinuation from the study will be recorded in detail. All changes of clinical laboratory parameters and vital signs at each scheduled time points from the baseline will be summarized according to treatment groups.

9.4.4 Exploratory analysis

The exploratory endpoints mainly focus on the add on effects of vitamin D3, so analysis are between vitamin D3 + saxagliptin + metformin (and insulin) group vs. saxagliptin + metformin (and insulin) group, including C–peptide changes during MMTT, HbA1c, FPG, PP and insulin-sparing effects and GADA titers. The relationship analysis of HLA-DQ with therapeutic effects will be applied to all three groups. Measurement data will be presented as mean, standard deviation, median, quartile, maximum value and minimum value. One-way ANOVA will be used to examine the baseline data and the changes in AUC during MMTT, FPG, PPG, HbA1c and insulin dose between groups. The changes of secondary variables describes above from the baseline within the same group will be analyzed with repeated measurement analysis of variance. The frequency counts and percentages will be summarized on proportion of patients achieving β-cell function protection and glycaemic control. Analyses in terms of age, BMI, baseline HbA1c, HLA-DQ genotypes, variations of immune repertoire before and after treatment, gender and other items will be collected.

10. STUDY AND DATA MANAGEMENT

10.1 Monitoring of the study

During outpatient visits of the study, the monitors will have regular contacts with investigators and designated staff by telephone, mail and/or email. Designated monitors will have monitoring visits to each study site. Investigators should allow monitors to

check the clinical, laboratory and pharmacy equipment to ensure not to violate GCP and local regulations. All CRF and corresponding original medical records (source doncument) will be prepared well in order to be examined in regular inspection for sponsor representatives. During inspection, monitors will verify whether the investigational team is adhering to the protocoland that data are being accurately recorded. All records will be inspected by China Food and Drug Administration (CFDA) and other regulatory agencies

According to ICH E6, Section 1.52, source files include, but are not limited to the following documents:

- According to ICH E6, Section 1.52, source document includes but not limited to following files:
- Outpatient records, visit records and hospitalized records
- Copies of medical records by medical personnel which have been verified for the accuracy.
- Data recorded by automated instrument, for example, X-ray photographs and other reports of imaging examination (such as ultrasonogram, CT scan, MRI and other radiographs), ECG, cardio rhythm graphs, electroencephalogram, polysomnography, pulmonary function test (no matter how these be recorded, including microfilm and photographic plates)
- Questionnaires about pain, life quality and case history by subjects
- Telephone records
- Pharmacy distributive and inventory records by chemists or other study staff
- Laboratory results and other laboratory test results, for example, records of urine pregnancy test and urine indicator paper test
- Messages or memorandum about treatment among doctors for sending to IRB/IEC
- Completed directly by subjects as a part of CRF of self source document (etc. questionnaires).

10.1.1 Source data

The medical record is considered as source material in a clinical trial and should be intact. The data in the Case Report Form derives from source material and should be in accordance with the latter. All observations and examination results in the trial should be recorded timely, accurately, intactly, standardly and factually on the medical record and filled correctly to the CRF. Alteration can not be made casually, if a error appears, correction must not interfere with the original note, name of whom and time should all be signed.

All laboratory data should be recorded during the study, the original report or its copy should be kept together with the research medical record. Data in the normal range should also be recorded in detail, remarkable deviation or clinical unacceptable data should be further verified. All examination results should indicate the unit of measurement.

10.1.2 Study agreements

The investigators at each centre should comply with all the terms, conditions, and obligations of the Clinical Study Agreement. In the event of any inconsistency between this Clinical Study Protocol and the Clinical Study Agreement, the terms of Clinical Study Protocol shall prevail with respect to the conduct of the study and the treatment of subjects.

Agreements between Principal Investigator and CRO and other investigators should be in place before any study-related procedures can take place, or subjects are screened.

10.1.3 Archiving of study documents

After completing the study, investigator should follow the principles outlined in the Clinical Study Agreement (CSA).

10.2 Study timetable and end of study

The end of the study is defined as the last visit of the last subject undergoing the study. The study is expected to start in Q1 2016 and to end by Q2 2019.

The study may be terminated at individual centres if the study procedures are not being performed according to GCP, or if recruitment is slow.

AstraZeneca and principal investigator may also terminate the entire study prematurely if concerns for safety arise within this study or in any other study with saxagliptin.

Upon terminating the study, the Principal Investigator/Investigator will report in writing the completion of the study as well as the summary of the results to the head of the study site in accordance with the study site’s rules.

10.3 Data management

During the implement and completion of the study, files of the study data will be stored by each study center. Paper editions of they study data should be stored in a locked cabinet.

The identity of each subject will be coded. Participation in this study is strictly confidential. Any information that is published will not reveal the identity of the subject. All records will be kept at minimum of 15 years by each study center.

Serious Adverse Event (SAE) Reconciliation

SAE Reconciliation Reports are produced and reconciled with Patient Safety database and/or the Investigational Site.

Management of external data

The patient's personal data, which are included in the investigator database, shall be treated in compliance with all applicable laws and regulations;

11. ETHICAL AND REGULATORY REQUIREMENTS

11.1 Ethical conduct of the study

This Clinical Trial will be conducted in accordance with the principles laid down by the 18th World Medical Assembly (Helsinki, 1964) and all applicable amendments laid down by the World Medical Assemblies, and the International Conference on Harmonisation

(ICH) guidelines for Good Clinical Practice (GCP). This clinical trial will be recorded in the public registry website clinicaltrials.gov before the enrolment of the first patient. The registry will contain basic information about the trial sufficient to inform interested patients (and their healthcare practitioners) how to enrol in the trial.

11.2 Subject data protection

The patient's personal data, which are included in the sponsor database, shall be treated in compliance with all applicable laws and regulations;

When archiving or processing personal data pertaining to the investigator and/or to the patients, the sponsor shall take all appropriate measures to safeguard and prevent access to this data by any unauthorized third party.

The sponsor also collects specific data regarding investigator as well as personal data from any person involved in the study which may be included in the sponsor’s databases, shall be treated by both the sponsor and the investigator in compliance with all applicable laws and regulations.

11.3 Ethics and regulatory review

This Clinical Trial will be conducted in compliance with national laws and regulations, as well as any applicable guidelines. An Ethics Committee should approve the final study protocol, including the final version of the Informed Consent Form and any other written information and/or materials to be provided to the subjects. The Investigator will ensure the distribution of these documents to the applicable Ethics Committee, and to the study site staff. The opinion of the Ethics Committee should be given in writing.

The Ethics Committee should approve all advertising used to recruit subjects for the study.

11.4 Informed consent

The Principal Investigator(s) at each centre will:

Ensure each subject is given full and adequate oral and written information about the nature, purpose, possible risk and benefit of the study

- Ensure each subject is notified that they are free to discontinue from the study at any
- time
- Ensure that each subject is given the opportunity to ask questions and allowed time to consider the information provided
- Ensure each subject provides signed and dated informed consent before conducting any procedure specifically for the study
- Ensure the original, signed Informed Consent Form(s) is/are stored in the Investigator's Study File
- Ensure a copy of the signed Informed Consent Form is given to the subject
- Ensure that any incentives for subjects who participate in the study as well as any provisions for subjects harmed as a consequence of study participation are described in the informed consent form that is approved by an Ethics Committee.

11.5 Changes to the protocol and informed consent form

Study procedures will not be changed without the mutual agreement of the sponsor and AstraZeneca.

If there are any substantial changes to the study protocol, then these changes will be documented in a study protocol amendment and where required in a new version of the study protocol (Revised Clinical Study Protocol). And the sponsor needs to ask approval from AstraZeneca.

The amendment is to be approved by the relevant Ethics Committee and if applicable, also the national regulatory authority approval, before implementation. Local requirements are to be followed for revised protocols. Sponsor will distribute any subsequent amendments and new versions of the protocol to each Principal Investigator(s). If local regulations require, any administrative change will be communicated to or approved by each Ethics Committee. The Investigator(s) may deviate from or make a change to the

protocol without documented agreement between the Principal Investigator and Sponsor only in the event of a medical emergency, e.g., it is only way to avoid an immediate hazard to the subjects. In such case, the Principal Investigator must notify details of the deviation or change, the reason, and a proposed revision in the protocol if required, to Sponsor and the head of the study site and via the head of the study site as soon as possible, in order to obtain their approval. A certificate of approval by the head of the study site as well as sponsor should be obtained

12. QUALITY CONTROL AND QUALITY ASSURANCE

Sponsor and investigator should perform their respective duties and comply with Clinical Trial Protocol to ensure quality control and quality assurance for clinical trial, using Standard Operation Procedure (SOP).

12.1 Monitoring

Monitor will prepared a complete monitoring plan before the initiation of the study, and during the study, the monitoring should be performed according to the monitoring plan. Sponsor is responsible for the monitoring of properly study performing and should ensure the consistency and availability of the data on CRFs by management and observation plan. The major duties of monitoring team are to assist investigators and sponsor to maintain the high quality in the aspects of ethics, scientificity, techniques and laws. In addition, monitoring team should ensure this study is curried out with the compliance of Clinical Trial Protocol, Chinese GCP and SOP.

During the monitoring of this study, the completeness and accuracy of the case record should be ensured, and should be verified with original record with the presence of investigators. The investigator will help with the performance of sponsor. During the monitoring of the study, monitoring team and sponsor will inspect: ICFs, recruitment and follow-up of subjects, the records and reports of SAEs, the distribution of investigational drugs, the protocol compliance for investigational drugs administration and dosing, and the accountability of investigational drugs, concomitant treatment and the quality of data.

12.2 Audits

The QA department of sponsor (or the third party) will perform audits for the study centres.

The auditor will perform systemic auditing for the activities and documents related to clinical study to assess whether the study comply with the protocol, SOP and other related regulations; whether the data is recorded truly, accurately, completely and without delay. The audits should be performed by the personnel unrelated to the clinical study.

12.3 Inspections

The authorities might perform inspections during or after the clinical study. As soon as the investigator is notified of a planned inspection by the authorities, he will inform the sponsor immediately. Sponsor will help and coordinate study centres to participate the inspection by authorities.

For the purpose of ensuring compliance with the Clinical Trial Protocol and applicable regulatory requirements, the investigator should permit auditing by or on the behalf of the sponsor and inspection by regulatory authorities. The investigator agrees to allow the auditors/inspectors to have direct access to his/her study records for review, being understood that these personnel is bound by professional secrecy, and as such will not disclose any personal identity or personal medical information.

The investigator will make every effort to help with the performance of the audits and inspections, giving access to all necessary facilities, data, and documents. As soon as the investigator is notified of a planned inspection by the authorities, he will inform the sponsor and authorize the sponsor to participate in this inspection. Any result and information arising from the inspections by the regulatory authorities will be immediately communicated by the investigator to the sponsor. The investigator shall take appropriate measures required by the sponsor to take corrective actions for all problems found during the audit or inspections.

13. LIST OF REFERENCES

[1].LI Yi-chong, LIU Xiao-ting, HU Nan, JIANG Yong, ZHAO Wen-hua. Disease burden on diabetes in China, 2010. Chinese Journal of Epidemiology, 2013,34(1): 33-6.

[2]. Leslie RD, Williams R, Pozzilli P. Clinical review: Type 1 diabetes and latent autoimmune diabetes in adults: one end of the rainbow. J Clin Endocrinol Metab 2006;91:1654-1659

[3]. Andersen MK, Lundgren V, Turunen JA, Forsblom C, Isomaa B, Groop P-H, Groop L, Tuomi T. Latent autoimmune diabetes in adults differs genetically from classical type 1 diabetes diagnosed after the age of 35 years. Diabetes Care 2010;33:2062-2064.

[4].Diabetes Society of Chinese Medical Association. Experts consensus on the diagnosis and treatment of LADA in China. Zhong Hua Tang Niao Bing Za Zhi, 2012,4:1-7

[5].Zhou Z, Xiang Y, Ji L, Jia W, Ning G, Huang G, Yang L, Lin J, Liu Z, Hagopian WA, Leslie RD. Frequency, Immunogenetics, and Clinical Characteristics of Latent Autoimmune Diabetes in China (LADA China Study) A Nationwide, Multicenter, Clinic-Based Cross-Sectional Study. Diabetes 2013;62:543-550

[6].Zhao Y, Yang L, Xiang Y, et al. Dipeptidyl peptidase 4 inhibitor sitagliptin maintains β-cell function in patients with recent-onset latent autoimmune diabetes in adults: one year prospective study. J Clin Endocrinol Metab. 2014;99:E876-880.

[7].Li X, Liao L, Yan X, et al. Protective effects of 1-alpha-hydroxyvitamin D3 on residual beta-cell function in patients with adult-onset latent autoimmune diabetes (LADA). Diabetes Metab Res Rev, 2009, 25:411-416.

[8].Miles J M, Rule A D, Borlaug B A. Use of metformin in diseases of aging[J]. Curr Diab Rep. 2014, 14(6): 490.

[9].Mei Z B, Zhang Z J, Liu C Y, et al. Survival benefits of metformin for colorectal cancer patients with diabetes: a systematic review and meta-analysis[J]. PLoS One. 2014, 9(3): e91818.

[10].Holman RR，Paul SK，Bethel MA．et a1．10-year follow-up of intensive glucose control in type 2 diabetes．N Engl J Med, 2008，359：1577-1589．

[11].Mu Y, Ji L, Ning G, et al.. Chinese experts consensus statement to metformin in the clinical practice[J].Chin J Diabetes,2014,22(8):673-681.

[12].Chinese Diabetes Society. Chinese Guideline for the Prevention and Management of Type 2 Diabetes (2013)[J]. Chin J Endocrinol Metab,2014,30(10):893-942.

[13].Kobayashi T, Maruyama T, Shimada A, et al. Insulin intervention to preserve β-cells in slowly progressive insulin dependent (type 1) diabetes mellitus. Ann N Y Acad Sci, 2002, 958:117-130

[14].Brophy S, Davies H, Mannan S, Brunt H, Williams R.Interventions for latent autoimmune diabetes (LADA) in adults. Cochrane Database Syst Rev. 2011;(9):CD006165.

[15].Barnett A H, Charbonnel B, Li J, et al. Saxagliptin add-on therapy to insulin with or without metformin for type 2 diabetes mellitus: 52-week safety and efficacy[J]. Clin Drug Investig. 2013, 33(10): 707-717.

[16].Sj√∂strand M, Iqbal N, Lu J, Hirshberg B. Saxagliptin improves glycemic control by modulating postprandial glucagon and C-peptide levels in Chinese patients with type 2 diabetes.Diabetes Res Clin Pract. 2014 ;105(2):185-91.

[17].Hyppönen E1, Läärä E, Reunanen A, Järvelin MR, Virtanen SM. Intake of vitamin D and risk of type 1 diabetes: a birth-cohortstudy. Lancet. 2001, 358(9292):1500-3.

[18].Gabbay M A, Sato M N, Finazzo C, et al. Effect of cholecalciferol as adjunctive therapy with insulin on protective immunologic profile and decline of residual beta-cell function in new-onset type 1 diabetes mellitus[J]. Arch Pediatr Adolesc Med. 2012, 166(7): 601-607.

[19].The Endocrine Society's Clinical Guidelines: Evaluation, Treatment, and Prevention of Vitamin D deficiency. J Clin Endocrinol Metab. 2011;96(7):1911-30.

[20].Maalouf J, Nabulsi M, Vieth R, et al. Short- and long-term safety of weekly high-dose vitamin D3 supplementation in school children[J]. J Clin Endocrinol Metab. 2008, 93(7): 2693-2701.

[21].Zhang X, Bao L,Bao Z, et al. Dosage research of serum 25-hydroxyvitamin D levels on different intervention treatments for children[J]. Chinese Journal of Clinical Pharmacology and Therapeutics,2011,16(12):1419-1422.

[22].Li J, Wang W. The comparison of vitamin D3 and alfacalcidol to treat hyperparathyroidism in patients withchronic kidney disease.[J].Chinese Community Doctors,2011,13(35):111-113.

[23].Zhen G,Zhang Y ,Qu D, et al. Effect of treatment of rickets with different dosage of vitamin D on eruption of deciduous teeth [J].Journal of Bengbu Medical College,2007,32(1):34-36.

[24].Ross AC, Taylor CL, Yaktine AL, et al. Dietary reference intakes for calcium and vitamin D. Washington, DC: The National Academies Press, 2011.

[25].Liao X, Zhang Z, Zhang H. Application guideline for vitamin D and bone health in adult Chinese (2014 Starter Edition). Chinese Journal of Osteoporosis. 2014,20(6): 718-722.

[26].Ning G Zhou Z. Endocrinology. Beijing: People's Medical Publishing House (PMPH), 2014, 136.

14. APPENDIX A SIGNATURES

Clinical Study Protocol

Drug Substance	saxagliptin and vitamin D3
Study Code	ISSSAXA0007
Edition Number	1.0
Date	29 Jul 2014

A randomized controlled、 open-label, multi-center study with 24-month fixed dose of saxagliptin and (or) vitamin D3 assessing protective effects on beta cell function in latent autoimmune diabetes in adults (LADA) treated with insulin

This Clinical Study Protocol and all Amendments to the CSP have been subjected to an internal AstraZeneca peer review.

I agree to the terms of this study protocol/amendment.

Principal Investigator: ____________ 29-07-2014

Zhiguang Zhou (Day month year)

Director, National Clinical Research Center for Metabolic Diseases

Head, Key Laboratory of Diabetes Immunology, Ministry of Education,

Central South University

139 Renmin Zhong Road

Changsha, Hunan 410011, P.R.China

(86)731-85292154

14. 附件

附件 1：

研究者签名页

方案编号：2015BAI12B13

研究题目：基于保护胰岛 β 细胞功能的 LADA 优化治疗方案多中心研究

——沙格列汀（和维生素 D3）联合二甲双胍（和胰岛素）治疗对 LADA 患者胰岛 β 细胞功能的影响：一项随机对照、开放的为期 104 周的多中心研究

版本号/日期：4.0 / 2016 年 01 月 01 日

我同意：

- 遵循研究方案中的规定以及ICH 和所有当地适用的药物临床试验质量管理规范（包括赫尔辛基宣言）开展此项试验。

我已经阅读了本方案的全文，同意所有内容。

颜湘	[signature]	2016.1.16
研究者姓名	研究者签字	日期

____
研究者职位

湖南省 长沙市
研究中心位置（省份，城市）

APPENDIX B: HOSPITALS LIST

No	Medical Centers
1	Peking University People's Hospital
2	Beijing Hospital
3	The First Hospital of China Medical University
4	The Second Hospital of Jilin University
5	Jilin Province People's Hospital
6	Jiangsu Province Hospital
7	The First Affiliated Hospital of Fujian Medical University
8	The Affiliated Hospital of Qingdao University
9	The First Affiliated Hospital of Chongqing Medical University
10	Xijing Hospital
11	Gansu Provincial Hospital
12	The First Affiliated Hospital of University of South China
13	Guangdong General Hospital
14	The First Affiliated Hospital of Shantou University Medical College
15	Heji Hospital Affiliated to Changzhi Medical College
16	The Second Xiangya Hospital of Central South University
17	Hainan General Hospital
18	The First Affiliated Hospital of Harbin Medical University
19	The First Affiliated Hospital of Guangxi Medical University
20	The First Affiliated Hospital of Guangzhou Medical University
21	Guangzhou First People's Hospital
22	The 2nd Affiliated Hospital and Yuying Children's Hospital of Wenzhou Medical University
23	The First Affiliated Hospital of Nanchang University

24	The Third Affiliated Hospital of Nanchang University
25	Henan Provincial People's Hospital
26	The First People's Hospital of Yunnan Province
27	The Second Hospital. University of South China
28	The First People's Hospital of Yueyang
29	The First People's Hospital of Changde City
30	Dongguan People's Hospital
31	Heping Hospital Affiliated to Changzhi Medical College
32	Quanzhou First Hospital
33	The Second Affiliated Hospital of Zhejiang University School of Medicine
34	Northern Jiangsu People's Hospital
35	Shanghai Xuhui District Central Hospital
36	The First People's Hospital of Huaihua
37	The First Affiliated Hospital of Henan University of Science and Technology
38	Tangshan Workers' Hospital
39	Affiliated Hospital of Inner Mongolia Medical University
40	Shenzhen Second People's Hospital

APPENDIX C:

Samples collection, transport and test results report procedures

(For Study 2015BAI12B13)

1. Sample Collection

1) Materials

1.1 Self-prepared: 5ml gel and vacuum blood collection tubes, 2ml and 5ml EDTA anticoagulated vacuum blood collection tubes, 2ml or 5ml sodium fluoride anticoagulated vacuum blood collection tubes, disposable venous blood collection needles, cuff, curved plate, skin disinfectant (75% alcohol or iodine) and sterilized cotton swab, etc.

1.2 Provided by center laboratory: Serum collection tubes, disposable blank label, small-sized sealing bags, large-sized sealing bags, disposable sterile Pasteur pipette, marking pen.

2) Test items

2.1 Items tested by each center: 0min, 60min, 120min blood glucose, HbA1c, blood routine, liver and kidney function, serum lipids, electrolytes, 24-hour urinary electrolytes (calcium and phosphorus), urinary albumin creatinine ratio.

2.2 Items tested by center laboratory:

a. GADA (sample should be collected under fasting status);

b. 25 (OH) D (sample should be collected under fasting status);

c. 0min, 60min, 120min C-peptide;

d. Genetic testing (for further research on HLA-DQ, DR, vitamin D receptor gene polymorphism and immune repertoire diversity, currently results of this part will not be provided).

3) Sample numbering and labeling

3.1 Screening numbering of subjects: L2-XX-YYY;

Notation: L2-fixed format, XX is the number of the center, YYY is the number of the subject (numbered sequentially according to the screening order);

Example: L2-20-001, "20" is the center numbered 20, "001" is the first subject during screening.

3.2 Sample numbering: screening number of the subject + V□ + a/c/d

Constitution: Screening number + times of visit + time points of blood collection should fill in the number of visits; a, c, d represents the time points of blood collection, which is 0min, 60min and 120min respectively.

Example of sample numbering: L2-20-001V5a, "L2-20-001" is the screening number, "V5a" is the 0min (fasting) sample of the 5th visit; similarly, the 60min postprandial sample of the 30th visit of the same subject should be L2-20-001V30c.

Labeling requirements (items tested by center laboratory): names, sample number of the subject should be written clearly on a blank label sticker and posted on the relevant serum collection tube.

4) Sample distribution and storage of center lab tested items

4.1 Fasting serum samples (GADA, 25 (OH) D, fasting C-peptide):

- Prepare one 5ml gel and vacuum blood collection tube;
- Collect 5ml fasting venous blood;
- Centrifuge at 2000g (approximately 3000 rpm / min) for 5 minutes to separate the serum within 2h;
- Use disposable sterilized Pasteur pipette and carefully transfer the serum into serum collection tube(s) with written label(s), confirm the lid is covered.

4.2 Fasting blood samples for further genetic research:

- Prepare 5ml EDTA anticoagulant tube, paste written label;
- Collect 3ml fasting venous blood;
- Immediately mix by inversion for more than 10 times.

4.3 60min, 120min postprandial C-peptide sample:

- Prepare 5ml gel and vacuum blood collection tube;
- 60min, 120min postprandial should each collect 5ml venous blood;
- Centrifuge at 2000g (approximately 3000 rpm / min) for 5 minutes to separate the serum within 2h;
- Use disposable sterilized Pasteur pipette and carefully transfer the serum into serum collection tube(s) with written label(s), confirm the lid is covered.

4.4 Storage:

- Specimens should be stored under -20 ℃ within 2h;
- If samples cannot be stored under -20 ℃ timely, then it should be stored at 4 ℃ temporarily (not more than 4h).

2. Transportation

1) Place the labeled serum collection tubes into relevant small-sized sealing bags, sealed.

2) Blood samples should all be put in a large-sized sealing bag while transportation to center laboratory each time, a printed list of names and sample numbers should be attached in the sealed bag, information of hospital and time of submission should be written on the large-sized sealed bag.

3) Deliver samples using dry ice to center laboratory every 14 days (refer to clinical sample transport operating instructions).

3. Results reporting

After receiving the samples, center laboratory will report the results of C-peptide and 25 (OH) D within a week, GADA results within 2 weeks.

Genetic testing result report is undetermined, currently after the completion of the study.

4. Center laboratory information

Center Laboratory Name: Key Laboratory of Diabetes Immunology, Ministry of Education

Central Laboratory Address: 18th Floor, Teaching and Science Building, The Second Xiangya Hospital, Wen Yi Street,, Fu Rong District, Changsha, Hunan Province

Postal Code: 410011
